# Self‐Assembled Nanostructured Microgels with Reconfigurable Morphologies

**DOI:** 10.1002/smll.202600013

**Published:** 2026-01-17

**Authors:** Cristina Álvarez‐Solana, Alberto Concellón, M. Blanca Ros

**Affiliations:** ^1^ Instituto de Nanociencia y Materiales De Aragón (INMA) CSIC‐Universidad De Zaragoza Zaragoza Spain; ^2^ Departamento de Química Orgánica Facultad De Ciencias Universidad De Zaragoza Zaragoza Spain

**Keywords:** bent‐core amphiphiles, complex emulsions, microgels, nanostructured materials, self‐assembly

## Abstract

We report a new class of supramolecularly assembled complex emulsions in which nanostructured organogels derived from bent‐core amphiphiles constitute one of the internal compartments. The molecular design of these amphiphiles, combining biphenyl (Bi) or ester (B1) lateral units with tetraethylene glycol and alkyl terminal chains, promotes efficient aggregation pathways and robust gelation. The resulting organogels exhibit fibrillar, tubular, and helical networks, with several systems displaying hierarchical arrangements characteristic of helical nanofilament (HNF) organizations, thereby transcribing liquid‐crystalline order into gel phases. Emulsification of the organogels into aqueous media yields supramolecular microgels that not only preserve their internal fibrillar architectures but also retain HNF‐like order, representing the first demonstration of nanostructured microgels formed through the self‐assembly of low‐molecular‐weight amphiphiles. Beyond single microgels, we prepare complex emulsions in which an organogel phase coexists with a fluorocarbon oil. These multicompartment droplets exhibit dynamic reconfigurability, switching between organogel‐in‐fluorocarbon‐in‐water (OG/F/W), Janus, and fluorocarbon‐in‐organogel‐in‐water (F/OG/W) morphologies. The ability to couple bent‐core self‐assembly with colloidal processing into reconfigurable complex emulsions establishes nanostructured microgels as an innovative adaptive soft‐material platform with opportunities in sensing, controlled delivery, bioimaging, and photonic technologies.

## Graphical abstract

This work presents bent‐core amphiphiles that self‐assemble into fibrillar, tubular, and helical nanofiber‐like nanostructures, forming organogels that can be emulsified into water as nanostructured microgels. These droplets preserve internal architectures and, when combined with fluorocarbon oils, yield multicompartment emulsions with dynamically reconfigurable morphologies, establishing a versatile supramolecular platform for adaptive soft materials in sensing, delivery, bioimaging, and photonics.

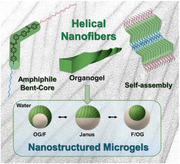

## 1 Introduction

Emulsions, which are dispersions of immiscible liquids stabilized in the form of droplets, are widespread in consumer products ranging from foods to pharmaceuticals, but they have also emerged as versatile frameworks for advanced functional materials [1, 2, 3, 4, 5, 6]. Their performance is closely tied to droplet geometry and interfacial composition, and the ability to manipulate internal structure has made emulsions valuable in controlled release, sensing, and optical applications. In recent years, attention has shifted from single emulsions to complex emulsions containing two or more phase‐separated liquids within the same droplet. These multiphase systems, which can adopt morphologies such as core–shell, Janus, or multiple‐internal droplets, allow spatial compartmentalization on the microscale and enable functions that cannot be achieved in single droplets [7]. Notably, dynamically reconfigurable complex emulsions have been introduced in which droplet morphology can be reversibly switched by changing surfactants, temperature, light, or other external cues [8]. Such adaptive droplets are being explored for tunable microlenses and ultrasensitive biosensing, underscoring the potential of complex emulsions as responsive soft matter platforms [9, 10, 11, 12, 13, 14].

Despite this progress, most complex emulsions rely on isotropic liquids, which restricts the degree of order and responsiveness that can be encoded in the droplet interior. A promising strategy to overcome these limitations is to incorporate liquid crystals (LCs). LCs are anisotropic fluids that combine long‐range molecular order with dynamic responsiveness to stimuli [15, 16], and their confinement within droplets gives rise to rich internal configurations and enables striking examples in optics, sensing, and active matter [17, 18, 19]. However, alternative classes of self‐assembled soft materials have scarcely been explored in this context, despite offering complementary opportunities. In particular, supramolecular organogels represent an attractive alternative: they combine the reversibility and adaptability of noncovalent interactions with the ability to generate extended fibrillar and tubular networks, often with long‐range internal order [20, 21, 22, 23]. Embedding such nanostructured gels inside droplets could provide a new pathway to microgels that are dynamic, reconfigurable, and structurally complex, yet no examples have been reported to date.

Bent‐core amphiphiles are uniquely suited to address this challenge [24, 25, 26], as their angular geometry enforces anisotropic packing with versatile supramolecular abilities to promote hierarchical assemblies such as helical nanofilaments and helical tubes, while their amphiphilic design allows them to be readily processed from solution and to form stable organogels across a range of solvents [27, 28, 29, 30, 31, 32]. Importantly, these same packing principles can also operate under the aforementioned solvent‐mediated conditions, enabling bent‐core amphiphiles to display a dual behavior, exhibiting liquid crystalline order in the bulk while forming anisotropic supramolecular assemblies in solution [33]. Such bent molecular architectures are well known to promote unconventional mesophases as a direct consequence of their geometry [32, 34, 35, 36, 37]. This molecular V‐shape favors polar packing, nanosegregation between rigid and flexible segments, and the formation of layered organizations, including B‐type phases in which each layer can carry a macroscopic dipole and associate into ferro‐ or antiferroelectric bilayers. Building on these features, we envisioned that bent‐core amphiphiles could enable the creation of supramolecular microgels that preserve the hierarchical order of their molecular building blocks while also being formulated into complex emulsions with reconfigurable morphologies, thereby expanding the scope of complex emulsions toward dynamic, nanostructured architectures.

**FIGURE 1:**
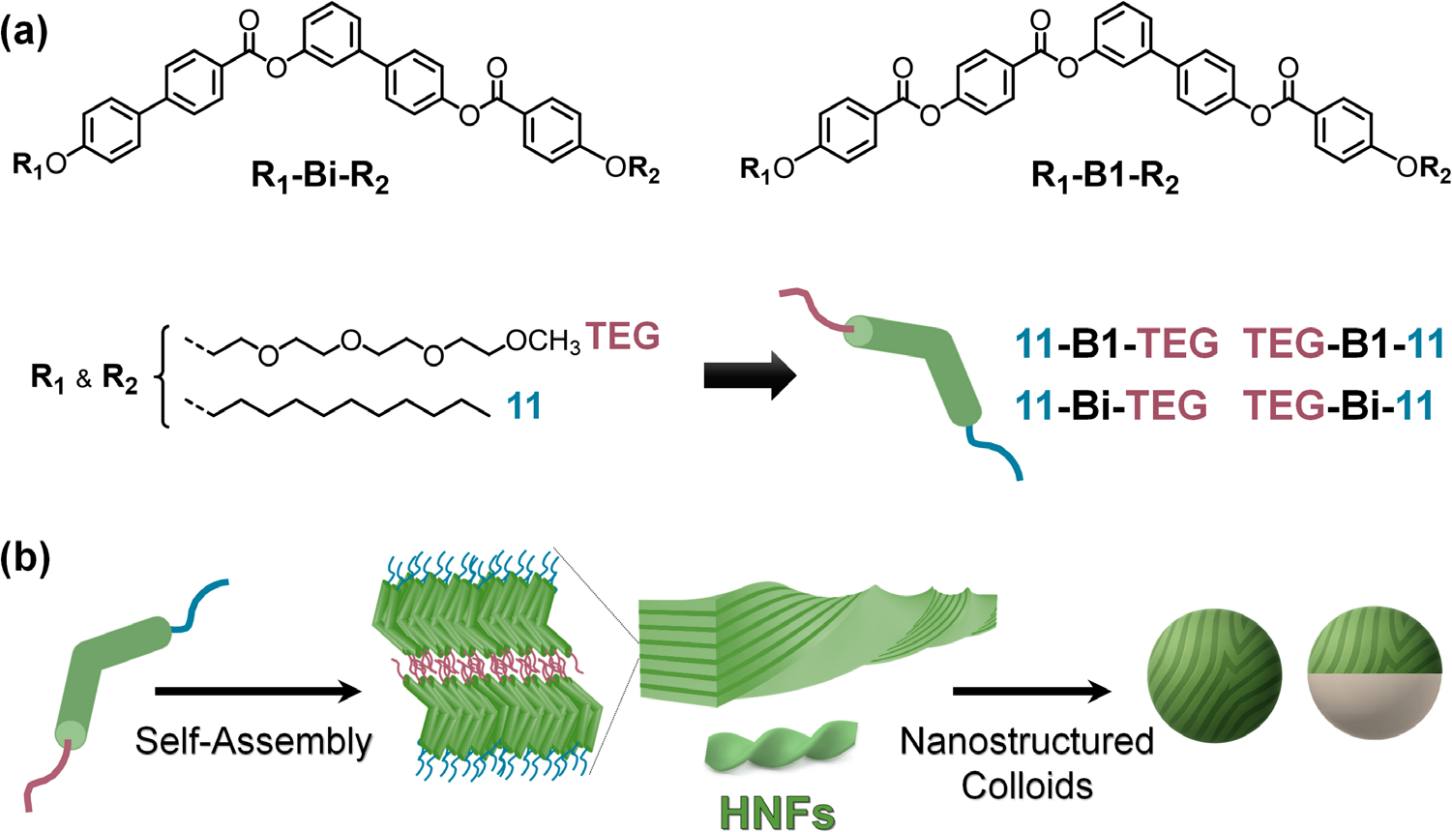
(a) Chemical structures of the four bent‐core amphiphiles (TEG‐B1‐11, TEG‐Bi‐11, 11‐B1‐TEG, and 11‐Bi‐TEG). (b) Schematic illustration of their hierarchical self‐assembly from molecular units into supramolecular fibers/tubes, helical nanofilament (HNF)‐type organizations, and ultimately organogel formation.

Herein, we report the realization of this concept through the design and synthesis of four amphiphilic bent‐core molecules that assemble into nanostructured organogels (Figure 1). Structural variations combining ester (**B1**) or biphenyl (**Bi**) lateral units [38] with hydrophilic tetraethylene glycol‐based (**TEG**) and hydrophobic alkyl (**11**) chains yield fibrillar and tubular supramolecular networks, including hierarchical helical nanofilament (HNF) organizations typical of some bent‐core liquid crystals. Emulsification of the gels into aqueous media produces discrete nanostructured microgels that preserve their internal fibrillar and HNF‐like architectures, thereby transcribing complex liquid‐crystalline order into colloidal droplets. Incorporation of fluorocarbon oils further enables the formation of complex emulsions whose morphology can be dynamically tuned among organogel‐in‐fluorocarbon‐in‐water (OG/F/W), Janus, and fluorocarbon‐in‐organogel‐in‐water (F/OG/W) structures by adjusting surfactant composition. This strategy establishes bent‐core amphiphiles as a versatile platform for functional supramolecular microgel emulsions, bridging molecular self‐assembly with responsive colloidal architectures, and suggests promising opportunities for photonic soft materials in which hierarchical and chiral supramolecular order can be confined, reconfigured, and functionally exploited at the microscale.

## 2 Results and Discussion

### 2.1 Synthesis and Structural Characterization

The synthesis of the asymmetric bent‐core amphiphilic compounds was accomplished following the synthetic strategies outlined in Schemes S1–S4. These synthetic routes were adapted and optimized from previously reported procedures involving related intermediates, starting from commercially available precursors [28]. All compounds were obtained in moderate to good yields and purified by standard chromatographic techniques. The purity and identity of the final products were confirmed by ^1^H and ^13^C nuclear magnetic resonance (NMR), infrared spectroscopy (IR), and high‐resolution mass spectrometry (HRMS). Full experimental procedures, including reaction conditions, purification steps, and spectral data for all intermediates and final compounds, are provided in the Supporting Information.

### 2.2 Self‐Assembly in Solution of the Bent‐Core Amphiphiles

The self‐assembly behavior in solution of the bent‐core (BC) amphiphiles was initially investigated through the analysis of their optical properties, taking advantage of the intrinsic fluorescence of the biphenyl moieties. Supramolecular aggregation is known to affect the photophysical properties of π‐conjugated systems due to changes in molecular packing, local polarity, and electronic interactions [39].

UV–vis absorption spectra of the compounds in THF (1 × 10^−5^ m) revealed absorption maxima in the range of 267–275 nm (Figure S18), which were used as excitation wavelengths for subsequent fluorescence measurements. Emission spectra were recorded for 1 × 10^−6^ m solutions in THF/H_2_O mixtures with water contents ranging from 0 to 90 vol%. For each measurement, the concentration was kept constant. THF efficiently solubilizes the aromatic bent‐core unit, while only the TEG chains exhibit affinity for water. Thus, these changes in solvent polarity and composition were used to induce amphiphilic self‐assembly [40].

**FIGURE 2:**
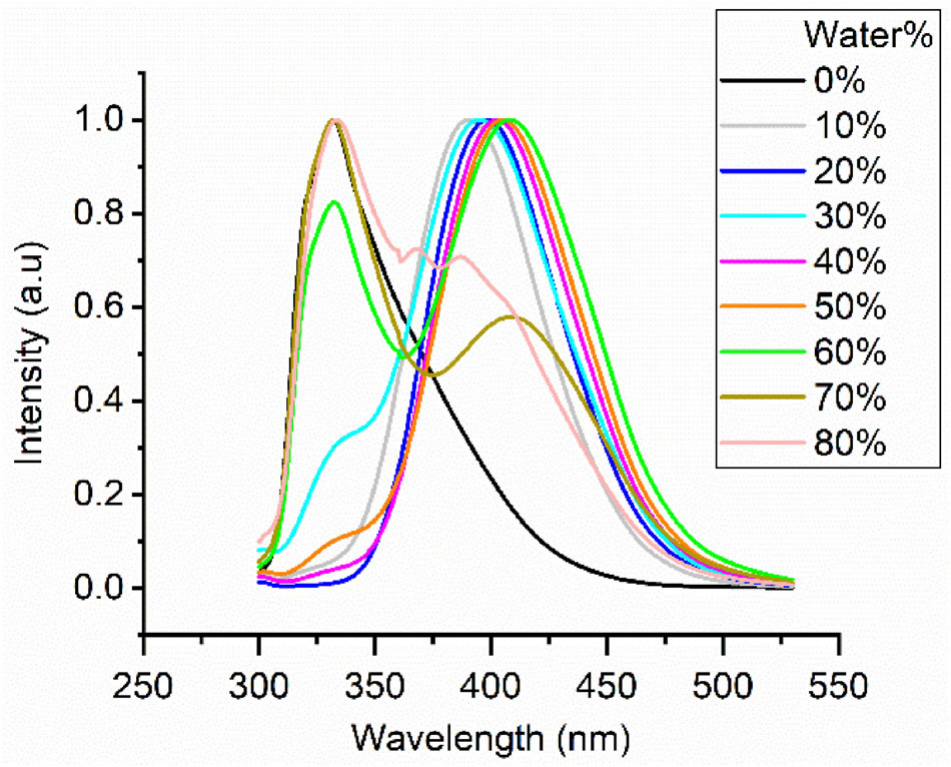
Evolution of the emission spectra of **11‐Bi‐TEG** in THF upon increasing water content (0–90 vol%) at an excitation wavelength of 267 nm showing quenching the emergence of a red‐shifted aggregate emission.

The emission of **11‐Bi‐TEG** showed a marked evolution with increasing water content (Figure 2). In pure THF, a strong band was observed at around 325 nm, which progressively decreased upon water addition. Simultaneously, a new emission band centered at around 410 nm appeared and intensified, consistent with the formation of aggregated species arising from π‐stacked biphenyl units. This red‐shifted emission cannot be attributed to classical biphenyl excimer formation, which is typically reported in the 350–365 nm range [41, 42], and therefore suggests the involvement of larger aggregated emissiv species. In contrast, isomer **TEG‐Bi‐11** displayed only modest spectral changes under identical conditions, limited to slight broadening and a minor bathochromic shift, indicating less efficient or disordered aggregation. These differences are attributed to the molecular architecture of the isomers. In **11‐Bi‐TEG**, the biphenyl groups are positioned adjacent to hydrophobic C11 chains, favoring close intermolecular packing and efficient π–π stacking. In contrast, in **TEG‐Bi‐11**, the biphenyl moieties are in proximity to hydrophilic TEG chains, which engage in strong interactions with water, reducing the probability of close π‐stacking. The **B1** isomers (**TEG‐B1‐11** and **11‐B1‐TEG**), which incorporate only a single biphenyl unit at the bent‐core junction, exhibited negligible fluorescence variation with increasing water content (Figures S19–S21). Even at 90 vol% water, only minor spectral broadening and red‐shifting were observed, suggesting a limited tendency to form π‐stacked aggregates relative to the **Bi** series.

**FIGURE 3:**
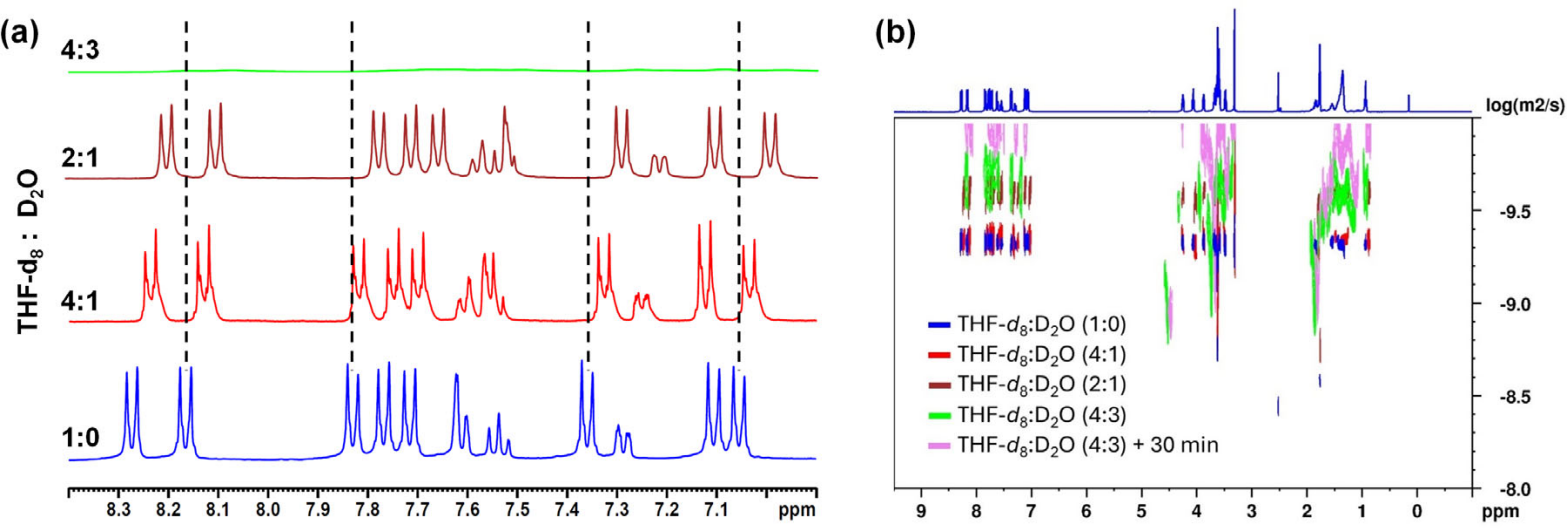
(a) ^1^H NMR spectra of the aromatic region of 11‐Bi‐TEG in THF‐d_8_/D_2_O mixtures with increasing water fractions, showing progressive upfield shifts and signal broadening associated with π–π stacking. (b) DOSY spectra of 11‐Bi‐TEG under the same conditions, evidencing reduced diffusion coefficients and the growth of supramolecular aggregates with increasing water content.

**FIGURE 4:**
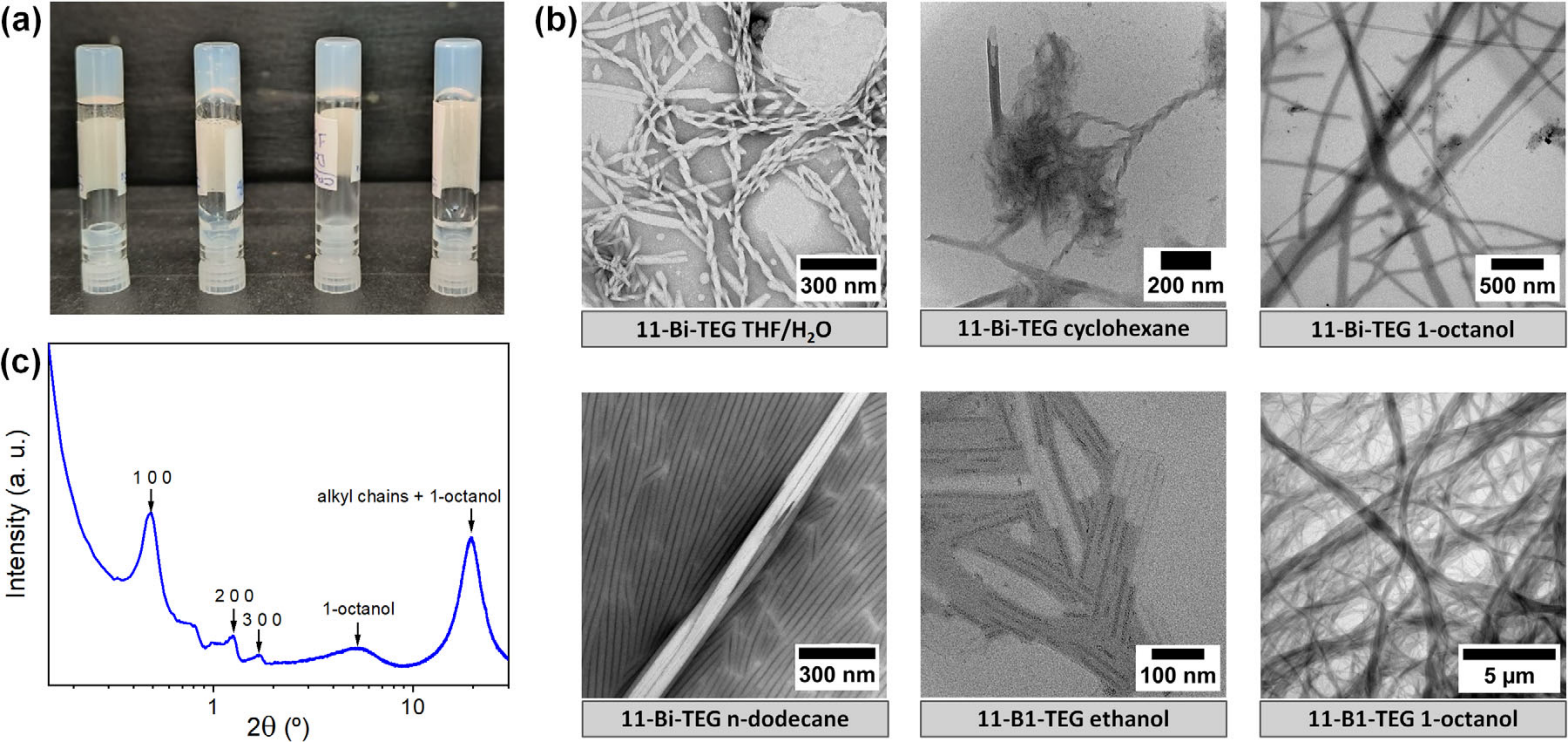
(a) Photographs of inverted vials showing gels of 11‐B1‐TEG in 1‐octanol (1 wt.%), TEG‐B1‐11 in 1‐octanol (1 wt.%), 11‐Bi‐TEG in n‐dodecane (1 wt.%), and TEG‐Bi‐11 in 1‐octanol (2 wt.%) (from left to right). (b) TEM images revealing fibrillar, tubular, and helical nanostructures characteristic of the supramolecular networks, highlighting representative morphologies observed in the gels. (c) XRD pattern of the 11‐B1‐TEG gel in 1‐octanol, showing lamellar order consistent with HNF‐type architectures.

To gain further insight into the aggregation mechanism, NMR experiments were performed in mixed THF‐*d*_8_/D_2_O solvents [43]. The study focused on **11‐Bi‐TEG** and **TEG‐Bi‐11** because of the pronounced differences observed in their emission properties. For **11‐Bi‐TEG**, progressive addition of D_2_O to a THF‐*d*_8_ solution induced a consistent upfield shift in the aromatic proton signals, attributed to π−π stacking interactions. These shifts intensified with increasing water content and were accompanied by notable signal broadening, both indicative of supramolecular aggregation in solution (Figure 3a). A particularly pronounced upfield shift was observed around 7.1 ppm, corresponding to aromatic protons adjacent to the hydrophobic alkyl chain. In contrast, the signal of the aromatic proton near the TEG chain remained essentially unchanged, likely due to its localization in a more hydrated, less tightly π−stacked environment. This difference suggests that the protons near the hydrophobic alkyl chains experience a stronger effect from the π−π stacking interactions, which increase their electronic shielding. At a THF‐*d*_8_/D_2_O ratio of 4:3 (v/v), all proton signals became significantly broadened, consistent with the formation of large supramolecular assemblies under these conditions. Notably, physical gelation under these conditions was observed approximately 30–45 min after water addition, indicating a strong tendency of the system to self‐assemble. TEM analysis of the resulting gel revealed an interconnected fibrillar network with a helical nanotubular morphology (Figure 4b), supporting the formation of self‐assembled nanostructures.

To gain insight into aggregate size, we performed ^1^H‐DOSY experiments—a powerful technique for estimating the sizes of supramolecular assemblies [43, 44]. The diffusion coefficients of **11‐Bi‐TEG** gradually decreased with increasing D_2_O content (Figure 3b), consistent with the formation of larger supramolecular aggregates driven by hydrophobic interactions and π–π stacking. In pure THF‐*d*_8_, the reduced diffusion coefficient suggested the presence of predominantly monomeric species. Upon increasing the water content, aggregate sizes increased. From diffusion data at 2:1 and 1:1 (v/v) THF‐*d*_8_/D_2_O mixtures, the supramolecular aggregates were estimated to consist of approximately eight molecules.

In contrast, **TEG‐Bi‐11** showed substantial signal attenuation and broadening upon water addition (Figure S9), and the complete disappearance of NMR signals was attributed to precipitation. No diffusion data could be obtained under these conditions. These findings underscore the higher aggregation capacity of **11‐Bi‐TEG** compared to its structural isomer. The observed differences can be rationalized by the spatial distribution of the biphenyl moieties within each molecule. In **11‐Bi‐TEG**, the proximity of biphenyl units to the hydrophobic chain favors close π–π stacking and ordered aggregation. Conversely, in **TEG‐Bi‐11**, hydration of the TEG chains inhibits the close biphenyl‐biphenyl interactions, hindering effective π–π interactions and suppressing supramolecular organization.

### 2.3 Gelation Properties of Bent‐Core Amphiphiles

The gelation capabilities of the synthesized amphiphilic BC‐molecules were systematically investigated across a range of solvents, including nonpolar solvents (n‐dodecane, cyclohexane, and toluene), aprotic polar solvents (1,4‐dioxane and DMF), and protic polar solvents (ethanol, 1‐octanol, and water). Physical organogels were prepared following a straightforward protocol: each compound was dissolved in the chosen solvent at 1 or 2 wt.% concentration, heated to approximately 5°C below the boiling point of the solvent, and then allowed to cool gradually to room temperature. Gelation was visually evaluated using the *vial inversion* method, where the formation of a stable gel is confirmed if the material does not flow upon inverting the vial (Figure 4a). The gelation results are summarized in Table 1. In addition, the gelation process was found to be fully reversible: upon heating, the gels underwent a gel–sol transition at a characteristic temperature, and upon cooling, gelation was reproducibly recovered, as evidenced by the sol–gel transition temperatures reported in Table 1.

**TABLE 1:** Gelation behavior of bent‐core compounds in solvents with different polarities, evaluated by the *vial inversion test*. All gels were prepared at a concentration of 1 wt.% except for 11‐Bi‐TEG in 1‐octanol. (G: Gel; I: Insoluble; S: Soluble). Sol–gel transition temperatures (°C) are shown in parentheses.

Compound	11‐B1‐TEG	TEG‐B1‐11	11‐Bi‐TEG	TEG‐Bi‐11
n‐Dodecane	I	S	G (55)	I
Cyclohexane	S	I ^b)^	G (50)	I
Toluene	S	S	S	S
1,4‐Dioxane	S	S	S	S
DMF	S	S	S	S
1‐Octanol	G (52)	I ^b)^	G (60) ^a)^	I
Ethanol	G (42)	I	I	I
Water	I	I	I	I

^a)^ Gel prepared at a concentration of 2 wt.%;

^b)^ Metastable gel that precipitates after 24 h of gel formation.

In solvents such as toluene, dioxane, and DMF, all compounds were completely soluble, and no gelation was observed. Nonetheless, the compounds were generally insoluble in water and ethanol, except for **11‐B1‐TEG**, which was the only molecule capable of forming a stable gel in ethanol at 2 wt.%. In 1‐octanol, **11‐B1‐TEG** formed stable gels at 1 wt.%, while **11‐Bi‐TEG** required 2 wt.% to achieve gelation. Among nonpolar solvents, cyclohexane and n‐dodecane exclusively induced stable gelation for **11‐Bi‐TEG**.

These results reveal a clear structure‐property relationship between the molecular design of the bent‐core amphiphiles and their gelation behavior. Gelation only occurs when the hydrophilic TEG chain is attached to the shorter segment of the bent‐core; compounds with TEG on the longer segment (**TEG‐B1‐11** and **TEG‐Bi‐11**) fail to form stable gels in any solvent. Among the gel‐forming compounds, **11‐B1‐TEG** shows strong gelation in polar solvents such as ethanol and 1‐octanol, while **11‐Bi‐TEG** gels in nonpolar solvents such as n‐dodecane and cyclohexane, and in 1‐octanol at higher concentrations. These observations suggest that efficient gelation arises from a delicate balance between hydrophilic and hydrophobic interactions, governed by the position of the TEG chain and the chemical nature of the longer segment. While TEG incorporation on the short segment enables the formation of supramolecular networks, the polarity, flexibility, and aromaticity of the longer lateral structure dictate the specific solvent environment in which fiber interweaving and network formation are most efficient.

To further elucidate the nanostructure of the gels, transmission electron microscopy (TEM) was carried out. Representative images (Figure 4b; Figure S22) revealed the formation of fibrous networks consisting of nanofibers with widths of a few nanometers and lengths extending to several micrometers. These fibers interconnect to form 3D networks that immobilize solvent molecules, resulting in gel formation. In some cases, lateral association of fibers into broader ribbon‐like aggregates was observed, although no chiral superstructures were detected. A summary of the observed morphologies and corresponding fiber dimensions is provided in Table S1. Briefly, in n‐dodecane, **11‐Bi‐TEG** formed gels composed of long fibers arranged into domains featuring helicoidal filaments, surrounded by solvent molecules. When cyclohexane was used as a solvent, this compound exhibited a mixture of helicoidal and tubular nanostructures. These TEM morphologies suggest that **Bi**‐type amphiphiles tend to form hierarchical supramolecular assemblies resembling the structures of helical nanofilaments characteristic of HNF‐based mesophases from bent‐core liquid crystals [27]. In 1‐octanol, long tubules are interconnected to form continuous networks. In contrast, **B1**‐type compounds primarily formed nanotubes of varying widths that aggregated into dense, fibrous networks.

Furthermore, to gain insight into the internal organization of these structures, small‐ and wide‐angle X‐ray scattering (SAXS and WAXS) experiments were performed. All diffraction patterns exhibited prominent peaks in the wide‐angle region that correspond to the characteristic distances of the solvent molecules (e.g., n‐dodecane or 1‐octanol) (Figure S24). These overlapped with broad halos and complicated the assignment of potential reflections from organized gel phases. However, in the small‐angle region, a series of periodic reflections indicative of lamellar ordering was observed across all samples. In certain cases ‐most notably for **11‐B1‐TEG** in 1‐octanol‐ reflections up to the fourth harmonic were detected, implying a lamellar arrangement with internal substructure (Figure 4c), characteristic of HNF‐like organizations. The calculated layer spacing is 182 Å, which is significantly larger than the length of the bent‐core molecule in its fully extended conformation (ca. 50 Å), suggesting a bilayer molecular arrangement combined with a high degree of solvent intercalation between adjacent layers in the gel state, as previously reported for related systems [28]. On this basis, a plausible supramolecular self‐assembly model can be proposed in which pairs of bent‐core amphiphiles associate through their aromatic cores to form the structural backbone of the assembly, while a thick interlamellar region is predominantly occupied by solvent molecules (Figure S25). Within this framework, the flexible tetraethylene glycol and alkyl chains extend into the surrounding solvent environment, and the resulting bilayer units can curl or wind to generate hollow tubular or helicoidal fibers. While alternative packing modes involving the association of multiple lamellar units cannot be excluded, the hierarchical and wound morphologies observed are fully consistent with self‐assembly pathways reminiscent of HNF‐type organizations reported for bent‐core LC phases.

### 2.4 Self‐Assembled Nanostructured Microgels

Microgels are gel particles of any shape with characteristic dimensions in the micrometer range, typically between approximately 0.1 and 100 µm [45]. Owing to their soft nature and internal network structure, microgels have attracted considerable interest in areas such as drug delivery, bioimaging, sensing, and adaptive soft matter [46, 47, 48]. While conventional microgels are typically composed of covalently crosslinked polymer networks, which are generally isotropic at the nanoscale, strategies to create physical microgels through the self‐assembly of low‐molecular‐weight compounds have, to our knowledge, remained largely unexplored. In this context, supramolecular microgels formed through non‐covalent interactions offer a unique opportunity to preserve the nanostructured, fibrillar, or even helical (chiral) organization of the parent gel within a micrometer‐sized object. Such internal nanostructuration fundamentally differentiates supramolecular microgels from conventional polymeric microgels and provides access to dynamic, reversible, and hierarchically ordered soft materials.

**FIGURE 5:**
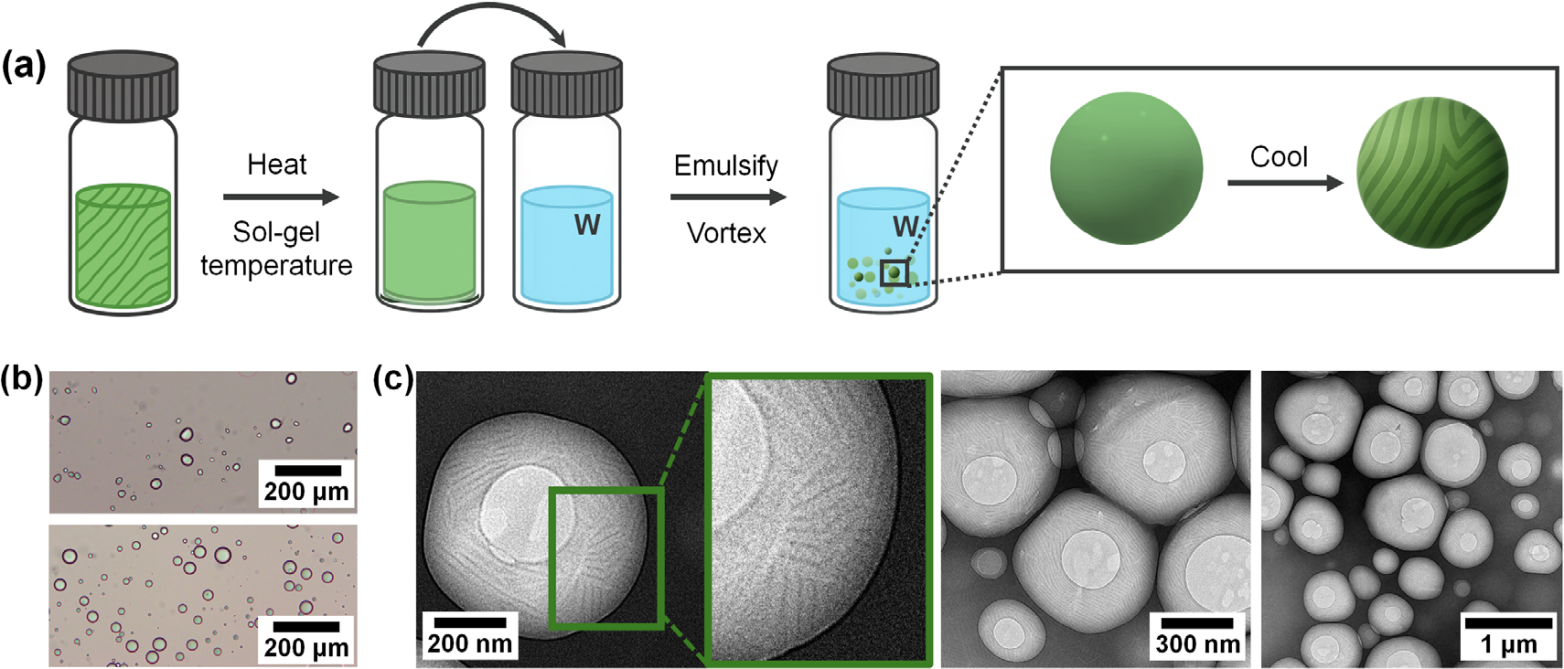
(a) Schematic representation of the preparation of single organogel‐in‐water microgel emulsions (b) Optical microscopy images of showing spherical to slightly anisotropic microgel droplets. (c) TEM images revealing that the microgels preserve a dense fibrillar/tubular supramolecular network (note that TEM was performed without staining nor coating, and the apparent holes within the droplets arise from beam‐induced damage of the organic material).

Initially, we prepared single emulsions (i.e., droplets with one phase‐separated liquids). Nanostructured microgels were obtained by dispersing the aforementioned hot gel solutions (above the sol–gel transition temperature) into an aqueous continuous phase to form single emulsions specifically, organogel‐in‐water (OG/W) systems. Upon cooling, these emulsions underwent gelation, yielding discrete, micrometre‐sized droplets containing self‐assembled nanostructures within (Figure 5a). Only organogel systems based on **11‐Bi‐TEG** were selected as the dispersed phase since this compound exhibited robust gelation behavior in solvents immiscible with water. Therefore, the dispersed phase consisted of **11‐Bi‐TEG** in 1‐octanol (2 wt.%), **11‐Bi‐TEG** in n‐dodecane (1 wt.%), **11‐Bi‐TEG** in cyclohexane (1 wt.%), whereas the continuous phase consisted of an aqueous solution of poly(vinyl alcohol) (PVA, 1 wt.%). A typical preparation involved adding 20 µL of the hot gel solution to 500 µL of the preheated PVA solution, followed by vigorous vortex mixing to generate the emulsion droplets. The emulsified mixture was then cooled gradually to room temperature to induce gelation within the droplets.

This procedure yielded stable microgel systems, whose morphology was evaluated by optical microscopy (Figure 5b). In all cases, the emulsions consisted of spherical or near‐spherical gelled droplets with diameters ranging from approximately 10 to 100 µm. Notably, the droplets prepared from 2 wt.% **11‐Bi‐TEG** in 1‐octanol exhibited a loss of sphericity, frequently adopting prolate or slightly anisotropic shapes. This deformation is likely the result of contraction during gelation, due to the higher concentration of the bent‐core gelator and the formation of a denser fiber network. The internal nanostructure of the microgel droplets was investigated by TEM (Figure 5c). The analysis revealed that each droplet contained a densely interconnected network of long fibrillar and tubular nanostructures. These architectures, formed via supramolecular self‐assembly of the bent‐core amphiphiles, effectively encapsulated the organic solvent and demonstrated the retention of nanostructured gel morphology within the microscale droplets. To the best of our knowledge, this constitutes the first example of nanostructured microgels formed through the self‐assembly of low‐molecular‐weight amphiphiles.

### 2.5 Complex Self‐Assembled Nanostructured Microgels with Tuneable Morphologies

**FIGURE 6:**
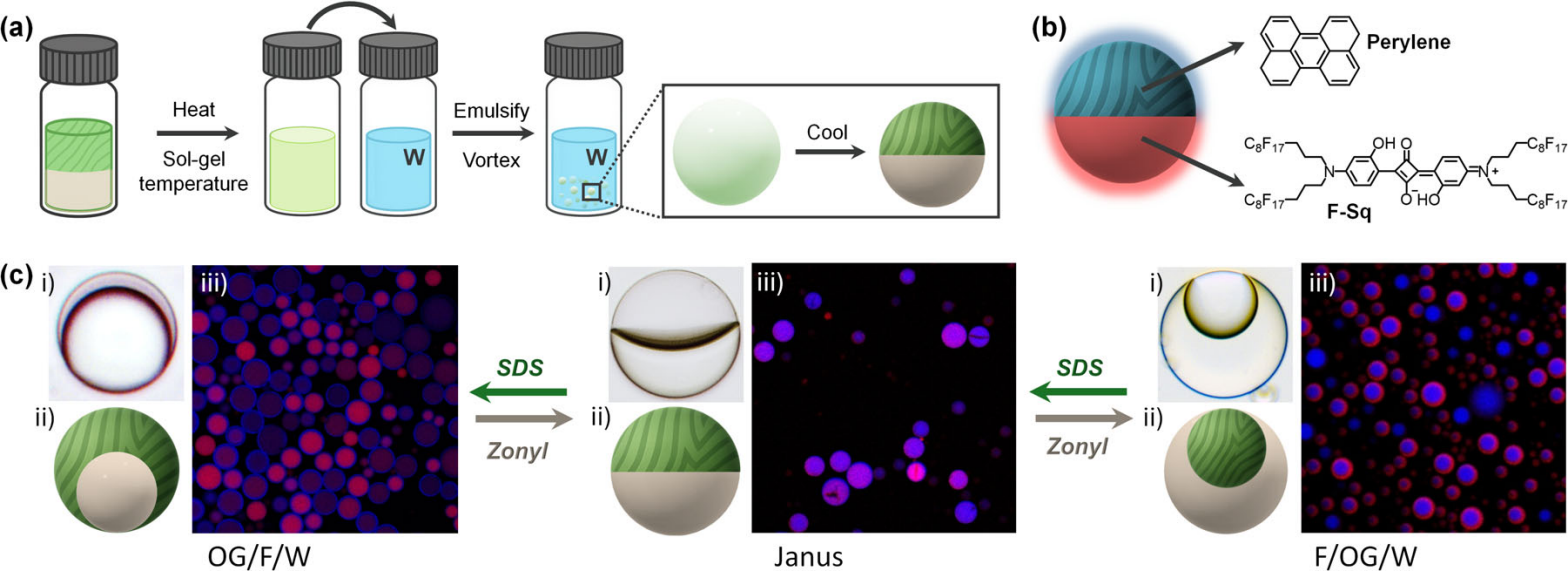
(a) Schematic representation of the preparation of complex microgel emulsions. (b) Chemical structures of the fluorescent dyes selectively incorporated into each compartment (blue‐emitting perylene in the organogel, red‐emitting fluorous squaraine in the fluorocarbon). (c) Dynamic reconfiguration of complex emulsion droplet morphology in response to the surfactant used (SDS vs. Zonyl), enabling reversible transitions between OG/F/W, Janus, and F/OG/W configurations: (i) side‐view optical microscopy image, (ii) schematic representation of the droplet morphology, and (iii) confocal fluorescence microscopy image.

To expand the structural and functional complexity of the nanostructured microgels, we prepared complex emulsions (i.e., droplets with two or more phase‐separated liquids) by incorporating a fluorinated oil as a second immiscible component within the dispersed phase [49, 50]. This led to the formation of multi‐compartment droplets, where one compartment consists of a nanostructured organogel formed by the self‐assembly of BC‐amphiphiles. Depending on the surfactant composition in the aqueous continuous phase, these droplets adopted tunable morphologies including organogel‐in‐fluorocarbon‐in‐water (OG/F/W), fluorocarbon‐in‐organogel‐in‐water (F/OG/W), and Janus morphologies (Figure 6a).

The formation of these emulsions was enabled by a temperature‐induced phase separation strategy. This approach relies on the existence of an upper critical solution temperature (UCST) between the organic and fluorinated components. Above this temperature, a homogeneous single phase is formed, which can be emulsified in water. Upon cooling, the components phase‐separate within the droplets, leading to internal compartmentalization. To apply this method, we first carried out solubility studies to evaluate the thermal miscibility of three selected gel‐forming solvents (1‐octanol, n‐dodecane, and cyclohexane) with two hydrofluoroether oils, namely HFE‐7200 and HFE‐7500. The goal was to identify combinations that are immiscible at room temperature but form a homogeneous mixture upon heating.

In the case of 1‐octanol, UCST values with either pure or mixed HFEs were above 70°C, making them unsuitable for practical processing. However, a 3:1 volumetric mixture of HFE‐7200:HFE‐7500 was compatible with n‐dodecane, while pure HFE‐7500 proved compatible with cyclohexane. These pairs were used to formulate the dispersed phase by mixing the organogel solvent containing the bent‐core amphiphile and the selected fluorinated oil in a 1:1 volume ratio. Emulsification was performed at elevated temperature (above the sol–gel transition and UCST) by vortexing 20 µL of the hot dispersed phase into 500 µL of an aqueous solution containing 1 wt.% surfactant. After emulsification, samples were cooled to room temperature to induce both phase separation and supramolecular gelation of the bent‐core amphiphile within the organogel compartment (Figure 6a).

The choice of surfactants in the continuous aqueous phase was found to be a critical factor governing the internal morphology of the resulting complex microgels (Figure 6b). When SDS, a hydrocarbon‐based surfactant, was used, the emulsions predominantly formed OG/F/W core–shell structures in which the nanostructured organogel encapsulated the fluorocarbon compartment. In contrast, emulsions stabilized with Zonyl FS‐300, a nonionic fluorosurfactant, exhibited inverted F/OG/W morphologies, with the fluorocarbon phase surrounding the organogel compartment. Intermediate mixtures of SDS and Zonyl yielded droplets with intermediate configurations, gradually transitioning from OG/F/W to Janus and finally to F/OG/W morphologies as the surfactant balance was adjusted. To probe the compartmental organization, fluorescent dyes were selectively incorporated into each phase: blue‐emitting perylene in the organogel and red‐emitting fluorous squaraine (**F‐Sq**) in the fluorocarbon oil, allowing direct visualization of the spatial separation of compartments by fluorescence microscopy (Figure 6b).

To investigate whether these nanostructured microgel droplets could undergo dynamic morphological reconfiguration, we performed surfactant‐exchange experiments. Owing to the physical gel nature of the organogel compartment, the supramolecular fiber network becomes mechanically locked upon cooling below the sol–gel transition temperature, thereby preventing morphological transitions of the droplets. Consequently, dynamic reconfiguration requires reheating the system above the sol–gel transition temperature to temporarily restore fluidity of the organogel phase. Under these conditions, exchanging the surfactant composition (e.g., switching from SDS to Zonyl or vice versa) alters the interfacial tension balance, driving reversible droplet morphology switching through sequential transformations from OG/F/W to Janus and ultimately to inverted F/OG/W configurations. These transitions were reproducibly observed by optical microscopy (Videos S1–S3). Complementary TEM analysis confirms that the microgel droplets exhibit a spherical morphology and contain an internal supramolecular network composed of interconnected fibrillar nanostructures (Figure S23).

## 3 Conclusion

In conclusion, we have demonstrated that BC‐amphiphiles are versatile building blocks for the formation of self‐assembled, nanostructured microgels with tunable complex morphologies. By combining biphenyl (**Bi**) lateral units with tetraethylene glycol‐based (TEG) chains positioned on the opposite lateral structure, we achieved efficient aggregation pathways and robust gelation, yielding fibrillar, tubular, and helical supramolecular networks. Importantly, several of these organogels exhibit hierarchical arrangements characteristic of helical nanofilament organizations similar to HNF‐type phases of bent‐core liquid crystals, thereby transcribing one of the most complex liquid‐crystalline architectures into gel phases. These nanostructured organogels provide the structural basis for translating molecular self‐assembly into colloidal formats.

Through emulsification into aqueous media, we generated nanostructured supramolecular single microgels that preserve their internal fibrillar and HNF‐like architectures within discrete micrometer‐sized droplets. To the best of our knowledge, this represents the first demonstration of physical (noncovalent) nanostructured microgels derived from the self‐assembly of low‐molecular‐weight amphiphiles, in contrast to conventional chemically crosslinked polymeric microgels. Beyond single microgels, we realized complex emulsions containing both an organogel compartment and a fluorocarbon oil. These multicompartment droplets display dynamic reconfigurability, switching between OG/F/W, Janus, and F/OG/W morphologies in response to surfactant composition, thus establishing a unique class of adaptive colloidal systems.

Overall, our findings highlight bent‐core amphiphiles as powerful molecular platforms for engineering nanostructured supramolecular gels and dynamically reconfigurable microgels. Beyond the formation of complex emulsions, this approach provides a fundamentally new strategy to confine and stabilize highly ordered LC‐like organizations—such as HNF arrangements—within discrete, micrometer‐sized droplets. In contrast to most previously reported LC emulsions, which predominantly rely on nematic or cholesteric phases with comparatively low internal complexity [17, 18, 19], the present system enables the formation of genuinely hierarchical and highly organized supramolecular nanostructures.

Moreover, the methodology developed for the preparation of these nanostructured microgels enables the incorporation of functional payloads, such as luminescent dyes, directly within the internal bent‐core supramolecular assemblies. This capability opens opportunities for templating and for the development of adaptive soft photonic systems in which optical functionality is encoded at the supramolecular level [51, 52, 53]. In this context, the coexistence of a fluorocarbon compartment introduces additional functionality, for example, by enabling total internal reflection at the organogel/fluorocarbon interface [10, 11], which could be exploited to enhance emission confinement and directionality. Importantly, the dynamic reconfiguration of droplet morphology provides a potential route to reversible on/off switching of optical responses. Future work will focus on leveraging this dynamic behavior to develop photonic microgels with programmable and reconfigurable optical functionalities.

Taken together, the combination of supramolecular nanostructuring, chirality, and reconfigurable compartmentalization positions these microgels as promising building blocks for advanced photonic materials, including light‐emitting systems, lasing media, and circularly polarized luminescence (CPL) emitters, as well as for broader applications in sensing, controlled delivery, and bioimaging.

## Funding

This work was financially supported by projects PID2023‐146811NA‐I00, PID2021‐122882NB‐I00, and PID2024‐156641NB‐I00, funded by MCIN/AEI/10.13039/501100011033 and by “ERDF: A way of making Europe”. A.C. acknowledges grant RYC2021‐031154‐I, funded by MICIU/AEI/10.13039/501100011033 and by the European Union NextGenerationEU/PRTR. Additional support was provided by the Gobierno de Aragón–FSE (research group E47_23R and PhD grant for C.A.‐S.).

## Conflicts of Interest

The authors declare no conflicts of interest.

## Supporting information




**Supporting File 1**: smll72399‐sup‐0001‐SuppMat.pdf.

**Supporting File 2**: smll72399‐sup‐0002‐VideoS1.mp4.

**Supporting File 3**: smll72399‐sup‐0003‐VideoS2.mp4.

**Supporting File 4**: smll72399‐sup‐0004‐VideoS3.mp4.

## Data Availability

The data that support the findings of this study are available in the supplementary material of this article.
