# Supplementary material for: Self‐Assembled Nanostructured Microgels with Reconfigurable Morphologies
Source: Small. 2026 Jan 17;22(15):e00013. doi: 10.1002/smll.202600013 (PMC12980474; doi:10.1002/smll.202600013)
Supplement: Supplementary file 1 — Supporting File 1: smll72399‐sup‐0001‐SuppMat.pdf. [file SMLL-22-e00013-s004.pdf]

Supporting Information

Self-Assembled Nanostructured Microgels with Reconfigurable Morphologies

Cristina Álvarez-Solana, Alberto Concellón, and M. Blanca Ros**

C. Álvarez-Solana, A. Concellón, M. B. Ros
Instituto de Nanociencia y Materiales de Aragón (INMA), CSIC-Universidad de Zaragoza, 50009 Zaragoza, Spain, Country
E-mail: aconcellon@unizar.es (A.C.), bros@unizar.es (M.B.R)

C. Álvarez-Solana, A. Concellón, M. B. Ros
Departamento de Química Orgánica, Facultad de Ciencias, Universidad de Zaragoza, 50009 Zaragoza, Spain.

Table of Contents

1. Materials and Methods S2
2. Experimental Procedures S4
3. Synthetic Procedures S5
3. NMR Spectra of Representative Compounds S9
4. FTIR spectrums S14
5. HRMS spectra S16
6. Absorption Spectra S20
7. Fluorescence Spectra S21
8.TEM Characterization S23
9.XRD S23
10. Supplementary Figures and Tables S28
11. Supplementary Videos S27
12. References S28

1. Materials and Methods

Chemical reagents used in this study were purchased from Aldrich and were used without further purification. Synthetic procedures and characterization data of the novel compounds are reported in section 2. Precursors **1**[1], **2**[2], **3**[3], **4**[4], **5**[5], **6**[6] and fluorous squaraine dye (**F-Sq**)[7] were synthesized adapting procedures already reported in the literature, and their characterization data agree with those previously reported, so the experimental details are not included.

Analytical TLC was performed on glass plate 60A coated with silica gel containing UV254 fluorescent indicator, 250 μm thick (Sigma-Aldrich). Column chromatography was carried out under flash conditions using 60 Å silica gel (Scharlab). NMR spectra were recorded using Bruker AV-400 spectrometers (operating at 400 MHz for ^{1}H and 100 MHz for ^{13}C) [Magnetic Resonance Service of CEQMA (UNIZAR-CSIC)]. Chemical shifts are reported in ppm relative to tetramethylsilane (TMS) and were determined using the residual solvent signal as the internal standard.

FT-IR spectra were recorded using Bruker Tensor 27 FTIR spectrometers [UNIZAR]. For solid products, KBr pellets were prepared with approximately 1% weight of the sample.

Mass spectral data were obtained using Bruker MicroTOF-Q and Bruker Esquire 3000+ spectrometers for ESI+ experiments [Mass Spectrometry Service of CEQMA (UNIZAR-CSIC)].

UV-vis spectra were obtained with a Cary 6000i UV–vis–NIR spectrophotometer [UNIZAR], using 10 mm wide HELLMA quartz cuvettes and a reference cuvette containing the same solvent. Fluorescence measurements were taken with a JASCO FP-8550 fluorescence spectrometer, using 10 mm wide HELLMA quartz cuvettes.

Nikon and Olympus BH-2 polarizing microscopes equipped with a Linkam THMS600 hot stage connected to a Linkam TMS94 temperature controller were used [UNIZAR]. The microscope is equipped with an Olympus DP12 camera controlled by Olympus DP-soft software. Measurements were performed using a Thermo Fisher Invitrogen Attofluor Cell Chamber.

Side-view images of the droplets were taken using a custom-built horizontal AmScope PZ200 polarizing microscope. For these experiments, emulsion droplets were deposited into a demountable quartz cuvette (path length: 100 μm) from Hellma.

The temperatures and enthalpies of the phase transitions were determined by calorimetric measurements using a TA Instruments Q20 system [Thermal Analysis Service of CEQMA (UNIZAR-CSIC)], with sealed aluminium capsules. Thermograms were recorded at a scanning

rate of 10°C/min, and the equipment was calibrated with indium. Thermogravimetric analyses were performed using a TGA Q5000IR thermobalance from TA Instruments [Thermal Analysis Service of CEQMA (UNIZAR-CSIC)], operating at a scanning rate of 10°C/min.

X-ray diffraction measurements were carried out using an XRD-PANanalytical Empyrean diffractometer ($CuK_{\alpha 1}$ = 1.5409 A) equipped with platform Scatter X78. The beam center and the *q* range were calibrated using the diffraction peaks of silver behenate. Samples were contained in Lindemann glass capillaries (0.9 mm diameter).

Confocal images were obtained using Zeiss LSM 700 laser scanning confocal at the Instituto Aragonés de Ciencias de la Salud (IACS). Measurements were performed using a Thermo Fisher Invitrogen Attofluor Cell Chamber.

2. Experimental Procedures

General procedure for the preparation of gel materials: into shell vials with polyethylene plug (40 mm length × 8.2 mm diameter, 1.00 mL capacity), variable amounts (1-7 mg) of the corresponding gelator and increasing amounts of solvent were placed. Then, these vials were gently heated with a heat gun until the solid material was completely dissolved, and homogeneous solution was achieved. The resulting isotropic solution was then cooled down in air to room temperature. No control over temperature rate during the heating-cooling process was applied. By the *inversion vial method*, the materials were classified as "gel" if it did not exhibit gravitational flow upon turning the vial upside-down at room temperature. The maximum concentration of these studies was 2% wt.

The morphological characterization of gels was carried out by transmission electron microscopy (TEM) recorded using a TECNAI G2 20 (FEI COMPANY) [LMA, Universidad de Zaragoza] operating at 200 kV (accelerating voltage). The samples were prepared by depositing one drop of a dispersion (lower concentrations than CGC) on a carbon film copper grid. The solvent was dried, with a piece of paper.

The emulsification procedure was carried out at temperatures above the sol–gel point to prevent the compounds from gelling. In the case of complex emulsions, slightly higher temperatures were reached, at which liquids formed a single phase. Emulsification was performed using a vortex mixer after adding 20 µL of the dispersed phase to 500 µL of the continuous phase. Once emulsified, the solution was allowed to cool to room temperature to facilitate gelation. CLASSIC vortex mixer from Velp® at 3000 rpm for 15 seconds.

The nanostructured gel morphology characterization of emulsions was carried out by transmission electron microscopy (TEM) recorded using a TECNAI G2 20 (FEI COMPANY) [LMA, Universidad de Zaragoza] operating at 200 kV (accelerating voltage). The samples were prepared by depositing one drop of the emulsion dispersion on a carbon film copper grid. The solvent was dried, with a piece of paper. For samples containing water, contrast was enhanced by staining with uranyl acetate. The sample was deposited onto a carbon-film copper grid following the same procedure as described above, and a 1–2% uranyl acetate solution was applied for 30–60 s. Excess stain was removed, and the grid was air-dried. Samples prepared in organic solvents were analyzed without staining.

3. Synthetic Procedures

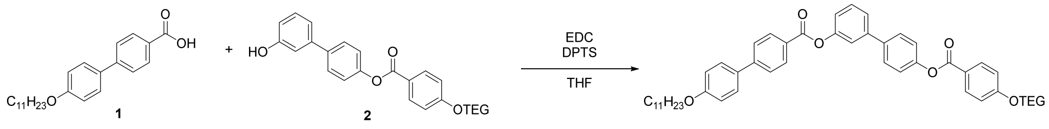

Scheme S1: *Reaction synthesis of* ***11-Bi-TEG****.*

4'-[(4-((2,5,8,11-tetraoxatridecan-13-yl)oxy)benzoyl)oxy]-[1,1'-biphenyl]-3-yl 4'-(n-undecyloxy)-[1,1'-biphenyl]-4-carboxylate [Compound 11-Bi-TEG]: In a flask, acid **2** (0.5 g, 1.5 mmol), compound **1** (0.37 g, 1 mmol), and DPTS (4-dimethylaminopyridinium p-toluenesulfonate) (0.11 g, 0.4 mmol) as a catalyst are solved in 10mL dry THF under Ar atmosphere and the mixture is cooled in ice bath. Later, EDC·HCl (0.29 g, 1.5 mmol) is slowly added. The reaction mixture is stirred for 24 hours. The white precipitate is filtered, and the resulting solution is evaporated to dryness. The obtained compound is purified by column chromatography (silica gel, eluent CH_2Cl_2:ethyl acetate 3:1), yielding a white solid. Yield: 0.3 g (36%).

^{1}H-NMR (400 MHz, $CDCl_3$): δ (ppm) = 0.89 (t, *J* = 6.4 Hz, 3H), 1.24-1.41 (m, 16H), 1.43-1.53 (m, 2H), 1.77-1.87 (m, 2H), 3.38 (s, 3H), 3.53-3.58 (m, 2H), 3.62-3.78 (m, 10H), 3.87-3.94 (m, 2H), 4.02 (t, *J* = 6.4 Hz, 2H), 4.20-4.26 (m, 2H), 6.97-7.05 (m, 4H), 7.21-7.26 (m, 1H), 7.27-7.32 (m, 2H), 7.44-7.49 (m, 1H), 7.49-7.54 (m, 2H), 7.57-7.63 (m, 2H), 7.63-7.68 (m, 2H), 7.68-7.74 (m, 2H), 8.13-8.19 (m, 2H), 8.23-8.30 (m, 2H). ^{13}C RMN (100 MHz, $CDCl_3$): δ (ppm) = 14.1, 22.7, 26.1, 29.3, 29.3, 29.4, 29.6, 29.6, 29.6, 32.0, 59.1, 67.7, 68.2, 69.6, 70.6, 70.7, 70.9, 72.0, 114.5, 115.0, 120.5, 121.9, 122.2, 126.6, 127.5, 128.3, 128.4, 129.8, 130.8, 132.3, 137.9, 142.1, 146.1, 150.8, 151.4, 159.6, 163.2, 164.9, 165.2. FTIR (KBr, ν: cm-1): 2919, 2853, 1733, 1606, 1294, 1267, 1254. ESI+ HRMS: m/z calcd $C_{52}H_{66}O_{10}N$ $[M+NH_4]^+$ 864.4681; found, 864.4683.

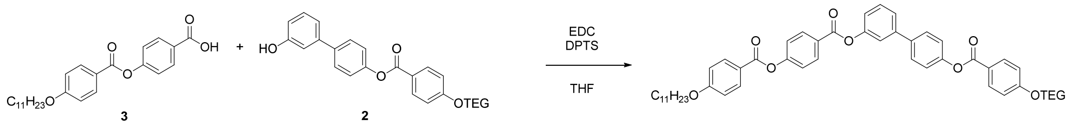

Scheme S2: *Reaction synthesis of* ***11-B1-TEG***.

4′-((4-((2,5,8,11-tetraoxatridecan-13-yl)oxy)benzoyl)oxy)[1,1′-biphenyl]-3-yl4-((4 (n-undecyloxy)benzoyl)oxy)benzoate [Compound 11-B1-TEG]: In a flask, acid **3** (0.32 g, 77 mmol), compound **2** (0.38 g, 0.77 mmol), and DPTS (4-dimethylaminopyridinium p-toluenesulfonate) (0.11 g, 0.4 mmol) as a catalyst are solved in 10mL dry THF under Ar atmosphere and the mixture is cooled in ice bath. Later, EDC·HCl (0.23 g, 1.19 mmol) is slowly added. The reaction mixture is stirred for 24 hours. The white precipitate is filtered, and the resulting solution is evaporated to dryness. The obtained compound is purified by column chromatography (silica gel, eluent CH_2Cl_2:ethyl acetate 7:3), yielding a white solid. Yield: 0.37 g (59%).

^{1}H-NMR (400 MHz, $CDCl_3$): δ (ppm) = 0.89 (t, J = 6.4 Hz, 3H), 1.22-1.41 (m, 16H), 1.42-1.53 (m, 2H), 1.78-1.88 (m, 2H), 3.38 (s, 3H), 3.53-3.57 (m, 2H), 3.62-3.73 (m, 8H), 3.73-3.77 (m, 2H), 3.88-3.93 (m, 2H), 4.06 (t, J = 6.4 Hz, 2H), 4.20-4.25 (m, 2H), 6.96-7.05 (m, 4H), 7.20-7.24 (m, 1H), 7.27-7.31 (m, 2H), 7.37-7.40 (m, 2H), 7.44-7.47 (m, 1H), 7.49-7.53 (m, 2H), 7.64-7.69 (m, 2H), 8.13-8.20 (m, 4H), 8.27-8.34 (m, 2H). ^{13}C RMN (100 MHz, $CDCl_3$): δ (ppm) = 14.3, 22.8, 26.1, 29.2, 29.5, 29.6, 29.7, 29.8, 29.9, 32.1, 59.2, 67.8, 68.5, 69.7, 70.7, 70.8, 70.9,71.1, 72.1, 114.6, 114.7, 120.6, 120.7, 121.1, 122.0, 122.3, 122.4, 124.8, 127.0, 128.4, 130.0, 132.0, 132.4, 132.6, 137.9, 142.3, 151.0, 151.5, 155.6, 163.3, 164.0, 164.5, 164.6, 165.0. FTIR (KBr, ν: cm-1): 3443, 3073, 2924, 2853, 1733, 1606, 1285, 1277, 1250. ESI+ HRMS: m/z calcd $C_{53}H_{66}O_{12}N$ $[M+NH_4]^+$ 908.4579; found, 908.4545.

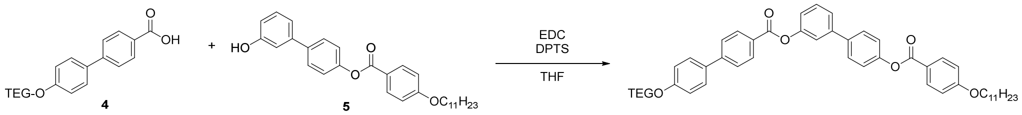

Scheme S3: *Reaction synthesis of* ***TEG-Bi-11****.*

4'-((4-(n-undecyloxy)benzoyl)oxy)-[1,1'-biphenyl]-3-yl 4'-((2,5,8,11-tetraoxatridecan-13-yl)oxy)-[1,1'-biphenyl]-4-carboxylate [Compound TEG-Bi-11]: In a flask, acid **4** (0.24 g, 0.58 mmol), compound **5** (0.27 g, 0.58 mmol), and DPTS (4-dimethylaminopyridinium p-toluenesulfonate) (0.07 g, 0.23 mmol) as a catalyst are solved in 10mL dry THF under Ar atmosphere and the mixture is cooled in ice bath. Later, EDC·HCl (0.17 g, 0.87 mmol) is slowly added. The reaction mixture is stirred for 24 hours. The white precipitate is filtered, and the resulting solution is evaporated to dryness. The obtained compound is purified by column chromatography (silica gel, eluent CH_2Cl_2:ethyl acetate 3:1), yielding a white solid. Yield: 0.26 g (53%).

^{1}H-NMR (400 MHz, $CDCl_3$): δ (ppm) = 0.89 (t, J = 6.4 Hz, 3H), 1.23-1.41 (m, 16H), 1.43-1.53 (m, 2H), 1.77-1.87 (m, 2H), 3.38 (s, 3H), 3.53-3.58 (m, 2H), 3.62-3.78 (m, 10H), 3.87-3.94 (m, 2H), 4.02 (t, J = 6.4 Hz, 2H), 4.20-4.26 (m, 2H), 6.97-7.01(m, 2H), 7.01-7.05 (m, 2H), 7.21-7.26 (m, 1H), 7.27-7.32 (m, 2H), 7.44-7.48 (m, 1H), 7.49-7.54 (m, 2H), 7.57-7.63 (m, 2H), 7.63-7.68 (m, 2H), 7.68-7.74 (m, 2H), 8.13-8.19 (m, 2H), 8.23-8.30 (m, 2H). ^{13}C RMN (100 MHz, $CDCl_3$): δ (ppm) = 14.1, 22.7, 26.0, 29.1, 29.3, 29.4, 29.6, 29.6, 29.7, 32.0, 59.1, 67.6, 68.4, 69.7, 70.5, 70.6, 70.9, 72.0, 114.3, 115.2, 120.5, 120.6, 121.4, 122.2, 124.6, 126.7, 127.5, 128.3, 128.4, 129.8, 130.8, 132.3, 132.4, 138.0, 142.3, 150.9, 151.4, 159.2, 163.6, 165.0. FTIR (KBr, ν: cm-1): 3445, 3068, 2919, 2853, 1733, 1606, 1294, 1267, 1254. ESI+ HRMS: m/z calcd $C_{52}H_{66}O_{10}N$ $[M+NH_4]^+$ 864.4681; found, 864.4690.

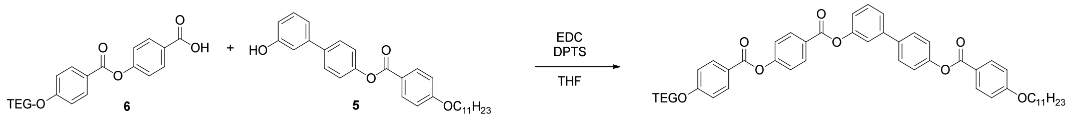

Scheme S4: *Reaction synthesis of* ***TEG-B1-11***.

3'-((4-((4-((2,5,8,11-tetraoxatridecan-13-yl)oxy)benzoyl)oxy)benzoyl)oxy)-[1,1'-biphenyl]-4-yl 4-(n-undecyloxy)benzoate [Compound TEG-B1-11]: In a flask, acid **6** (0.26 g, 0.58 mmol), compound **5** (0.27 g, 0.58 mmol), and DPTS (4-dimethylaminopyridinium p-toluenesulfonate) (0.87 g, 0.27 mmol) as a catalyst are solved in 10mL dry THF under Ar atmosphere and the mixture is cooled in ice bath. Later, EDC·HCl (0.2 g, 1 mmol) is slowly added. The reaction mixture is stirred for 24 hours. The white precipitate is filtered, and the resulting solution is evaporated to dryness. The obtained compound is purified by column chromatography (silica gel, eluent CH_2Cl_2:ethyl acetate 7:3), yielding a white solid. Yield: 0.27 g (52%).

^{1}H-NMR (400 MHz, $CDCl_3$): δ (ppm) = 0.89 (t, J = 6.4 Hz, 3H), 1.23-1.41 (m, 16H), 1.42-1.53 (m, 2H), 1.78-1.88 (m, 2H), 3.38 (s, 3H), 3.53-3.57 (m, 2H), 3.62-3.73 (m, 8H), 3.73-3.77 (m, 2H), 3.88-3.93 (m, 2H), 4.05 (t, J = 6.4 Hz, 2H), 4.20-4.25 (m, 2H), 6.96- 7.05 (m, 4H), 7.20-7.24 (m, 1H), 7.27-7.31 (m, 2H), 7.37-7.40 (m, 2H), 7.44-7.47 (m, 1H), 7.48-7.53 (m, 2H), 7.64-7.68 (m, 2H), 8.13-8.20 (m, 4H), 8.27-8.34 (m, 2H). ^{13}C RMN (100 MHz, $CDCl_3$): δ (ppm) = 14.1, 22.7, 26.0, 29.1, 29.3, 29.4, 29.6, 29.8, 29.9, 32.0, 59.1, 67.8, 68.5, 69.7, 70.55, 70.7, 70.8, 70.9,71.0, 72.0, 114.3, 114.6, 120.4, 120.6, 121.4, 121.5, 122.1, 122.2, 124.7, 126.9, 128.3, 129.9, 131.9, 132.3, 132.4, 137.8, 142.2, 150.9, 151.3, 155.4, 163.4 163.6, 164.28, 164.5, 165.0. FTIR (KBr, ν: cm-1): 3451, 3069, 2924, 2853, 1733, 1606, 1285, 1277, 1256. ESI+ HRMS: m/z calcd $C_{53}H_{66}O_{12}N$ [M+NH_4]+ 908.4579; found, 908.4631.

3. NMR Spectra of Representative Compounds

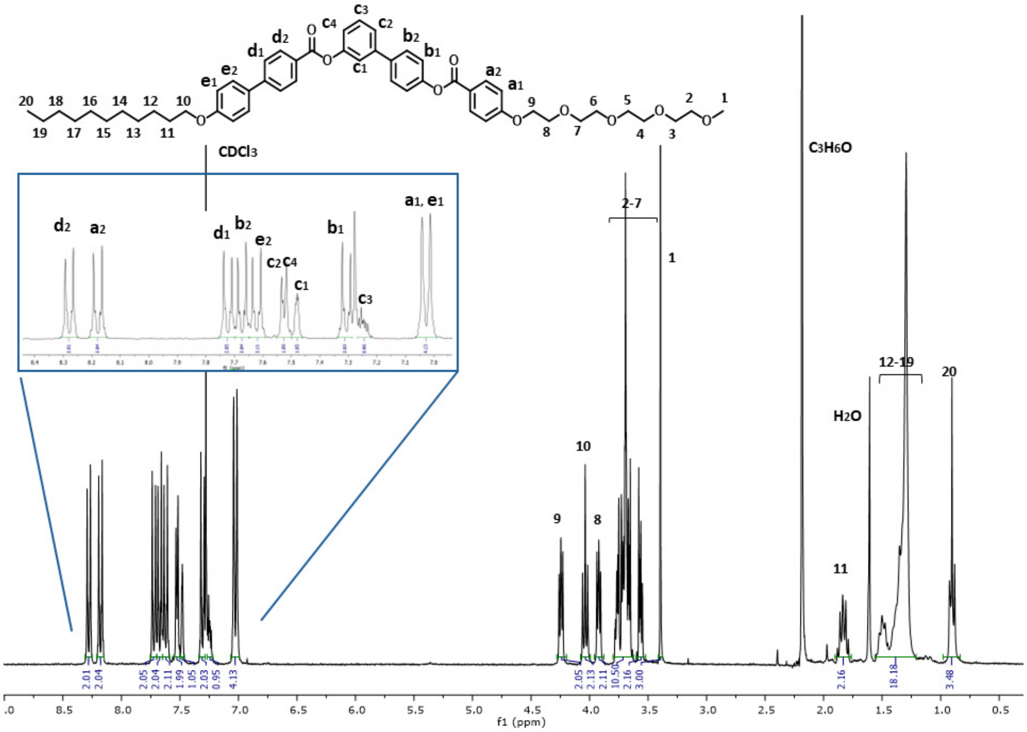

Fig. S1: *¹H NMR spectrum (400 MHz, CDCl₃, δ (ppm)) of the compound* ***11-Bi-TEG***

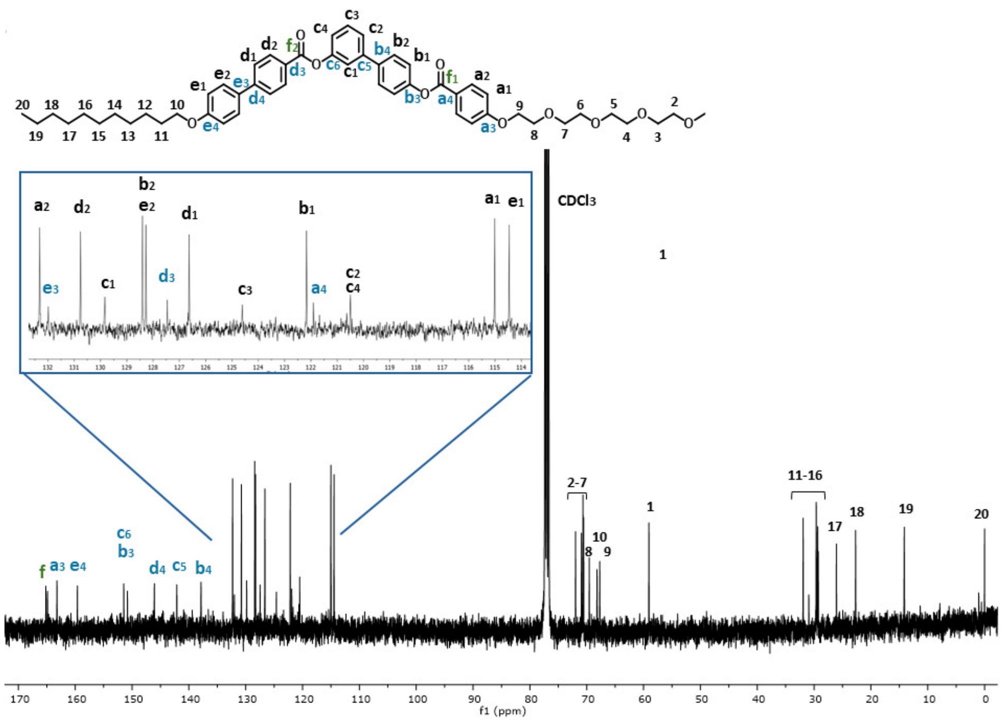

Fig. S2: *¹³C NMR spectrum (100 MHz, CDCl3, δ(ppm)) of the compound* ***11-Bi-TEG***

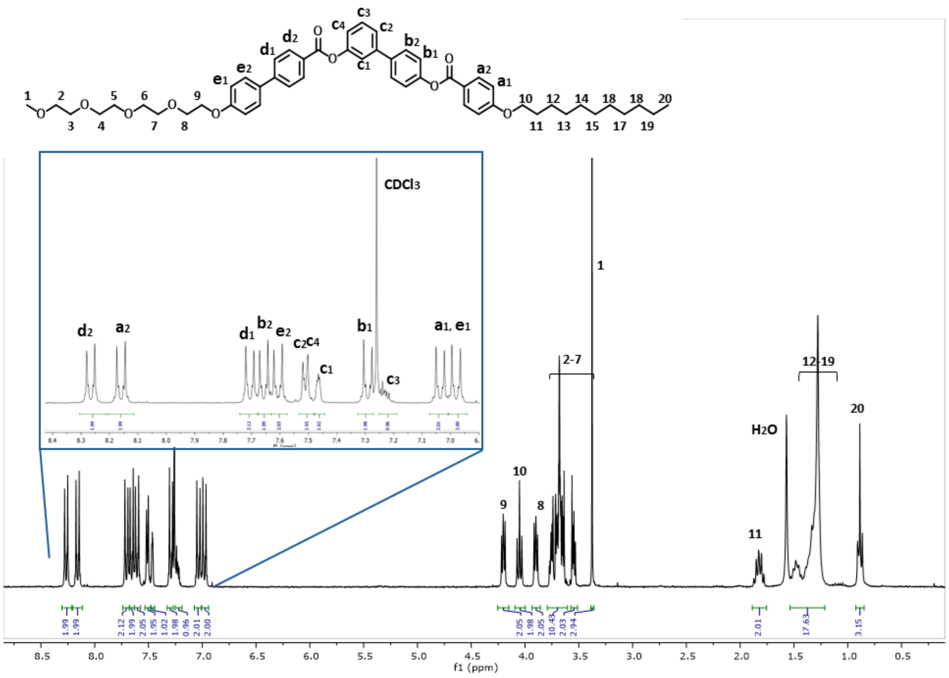

Fig. S3: *^{1}H NMR spectrum (400 MHz, $CDCl_3$, δ (ppm)) of the compound* ***TEG-Bi-11***

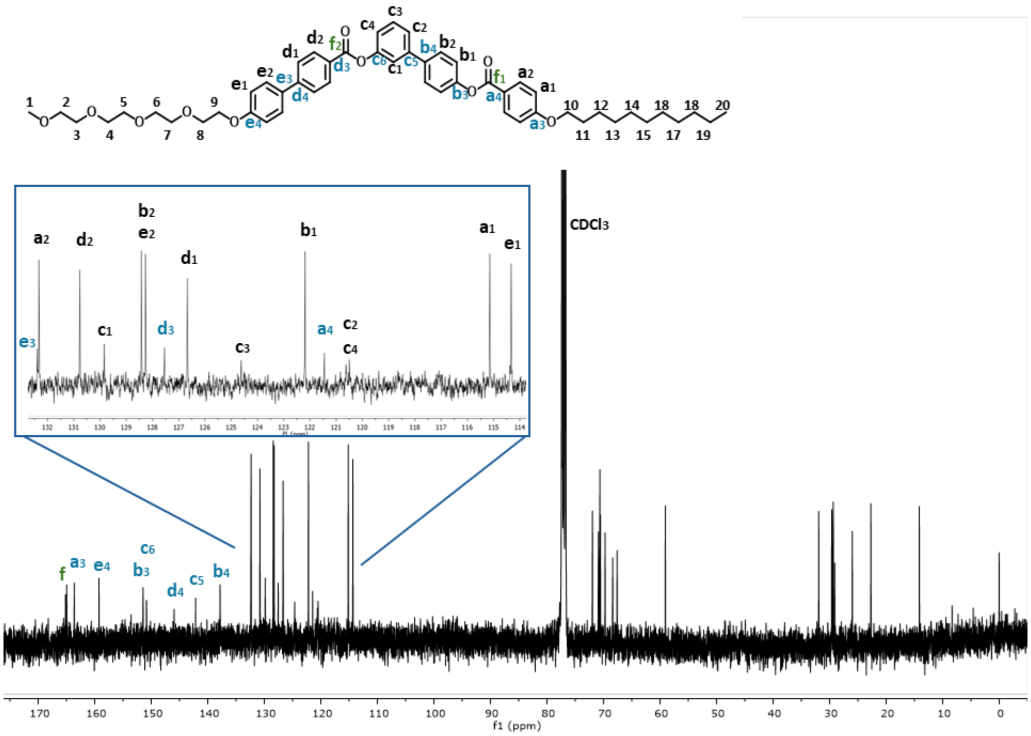

Fig. S4:

^{13}C NMR spectrum (100 MHz, CDCl3, δ(ppm)) of the compound ***TEG-Bi-11***

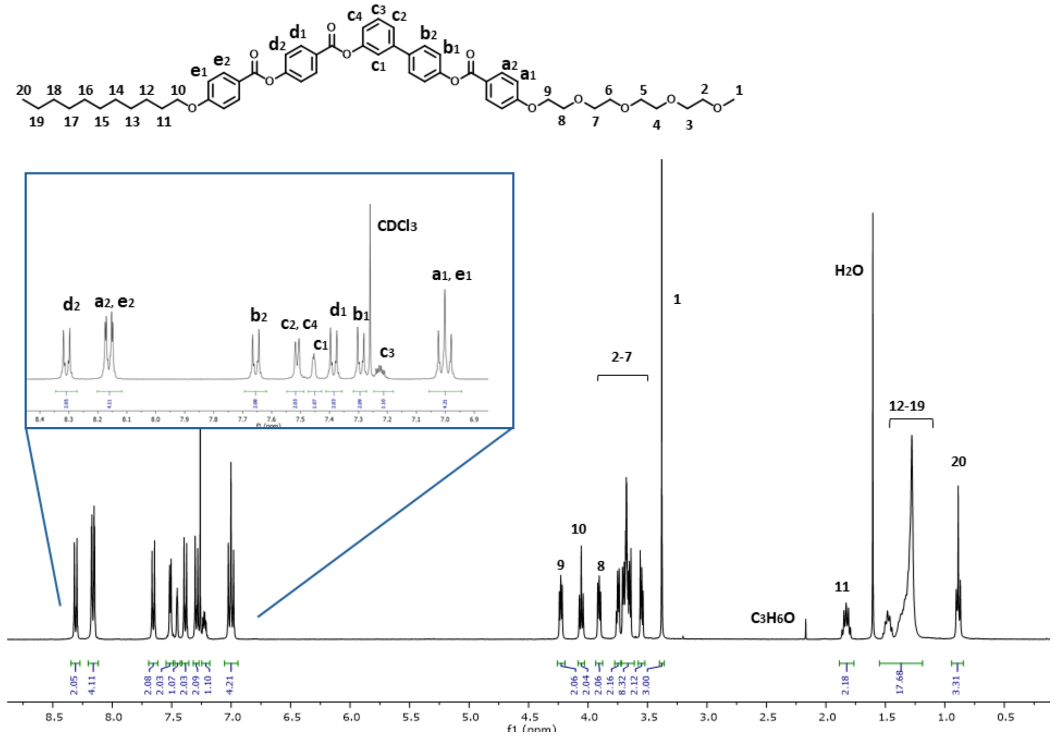

Fig. S5: *¹H NMR spectrum (400 MHz, $CDCl_3$, δ (ppm)) of the compound* ***11-B1-TEG***

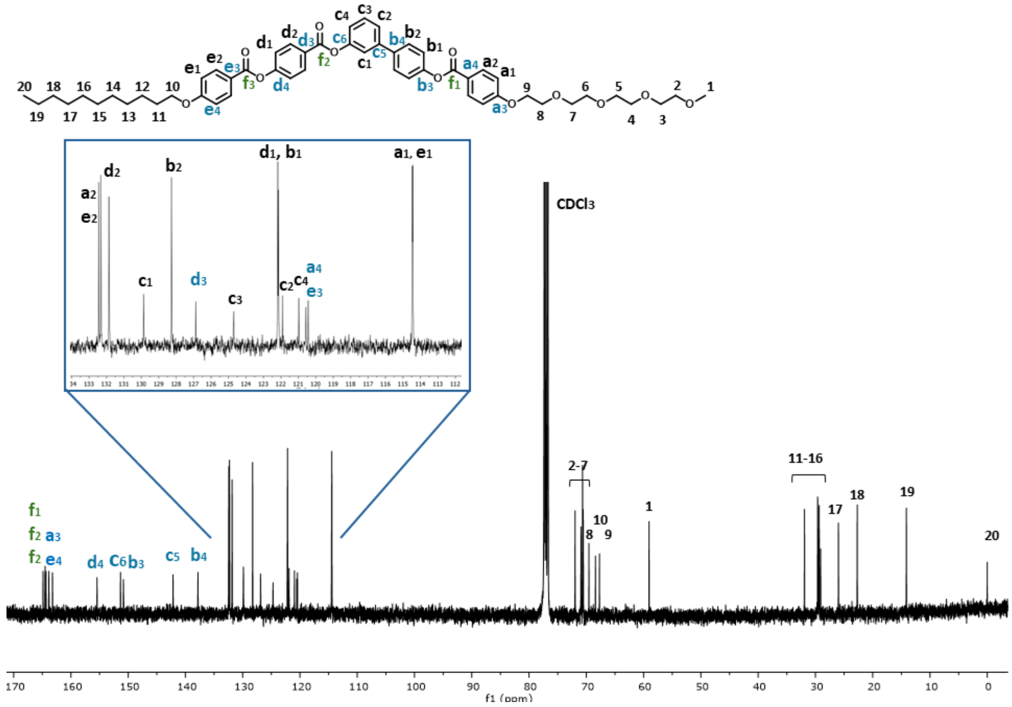

Fig. S6: *¹³C NMR spectrum (100 MHz, CDCl3, δ(ppm)) of the compound* ***11-B1-TEG***

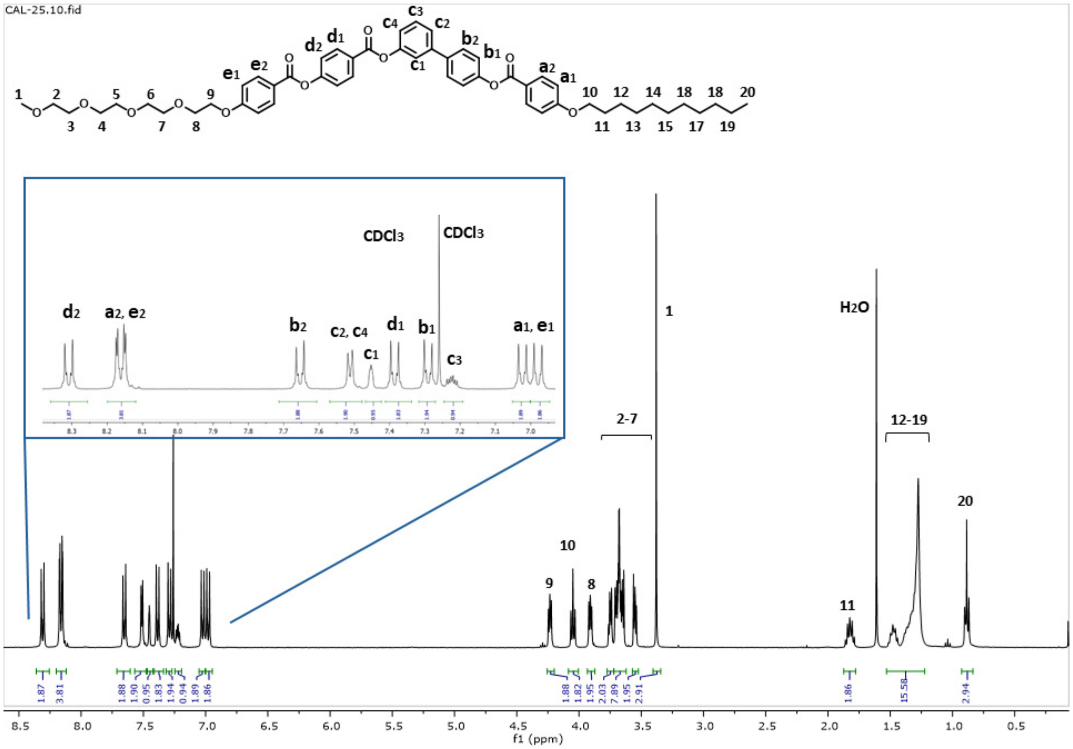

Fig. S7: *^{1}H NMR spectrum (400 MHz, $CDCl_3$, δ (ppm)) of the compound* ***TEG-B1-11***

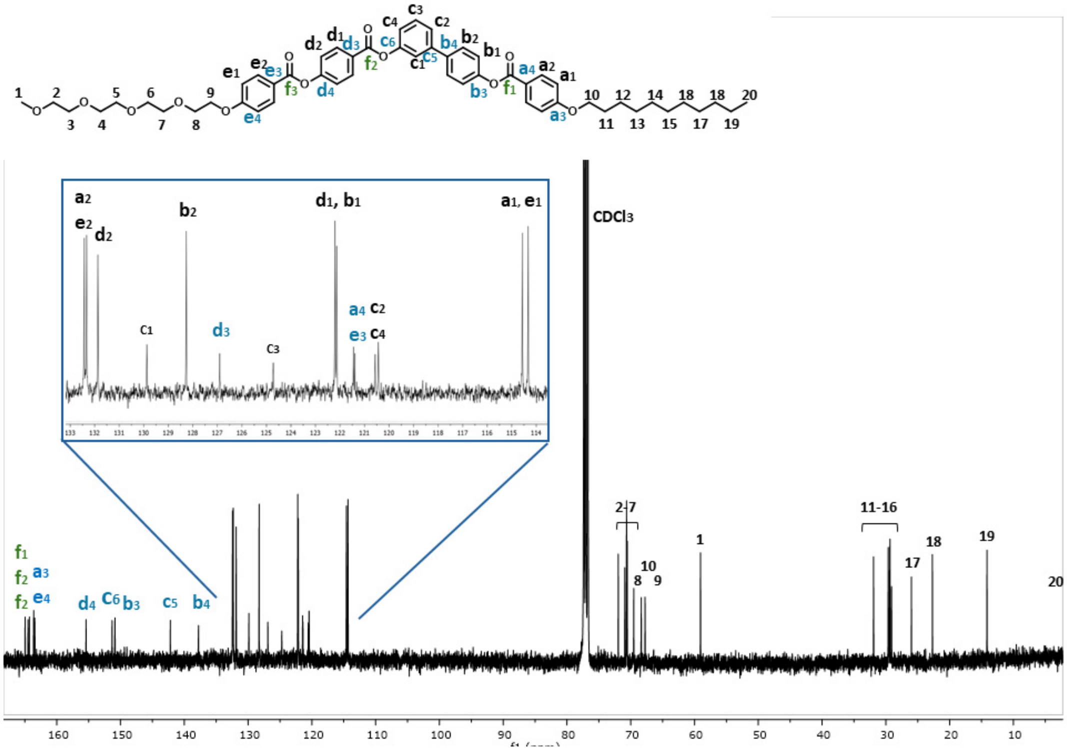

Fig. S8: *^{13}C NMR spectrum (100 MHz, CDCl3, δ(ppm)) of the compound* ***TEG-B1-11.***

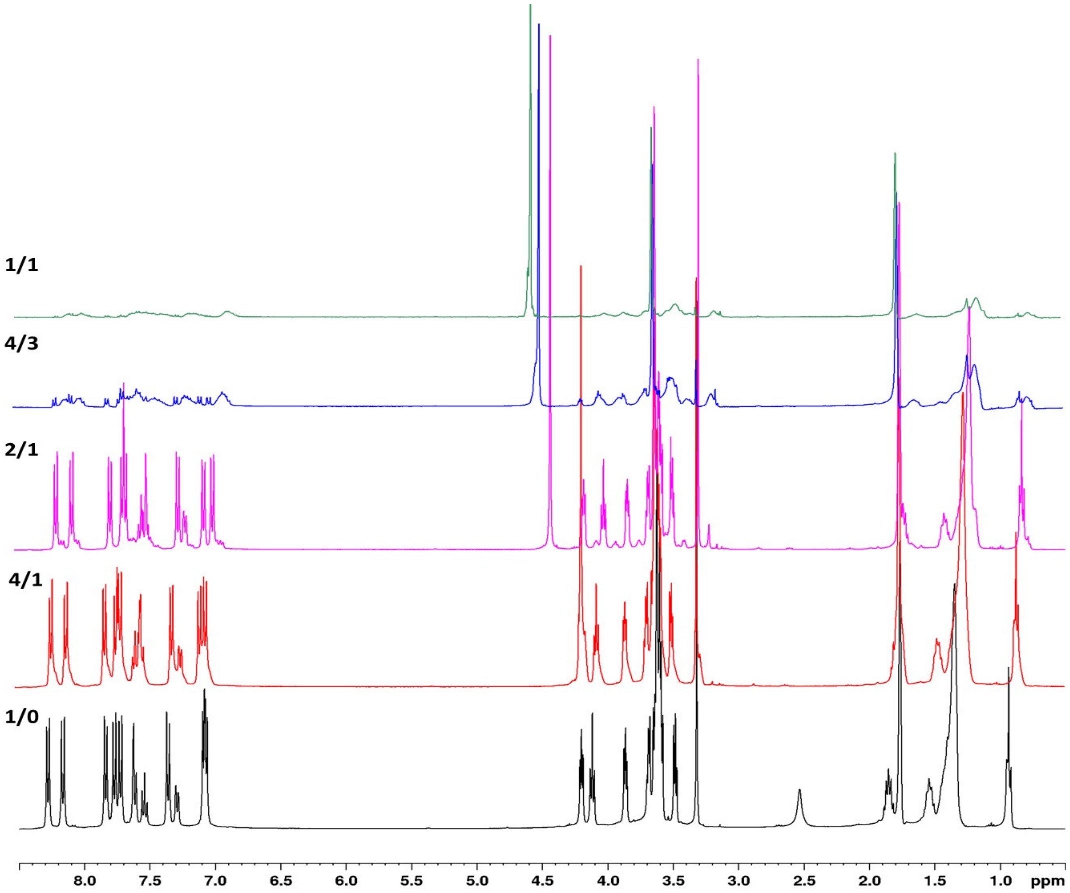

Fig. S9: *^{1}H NMR spectra (400 MHz, $CDCl_3$, δ (ppm) at different THF-d8/D_2O ratios of the compound* ***TEG-Bi-11***

4. FTIR Spectra

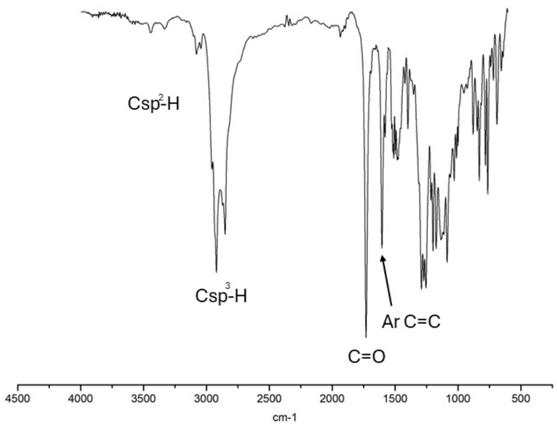

Fig. S10: *FTIR (KBr) spectrum of compound* ***11-Bi-TEG***

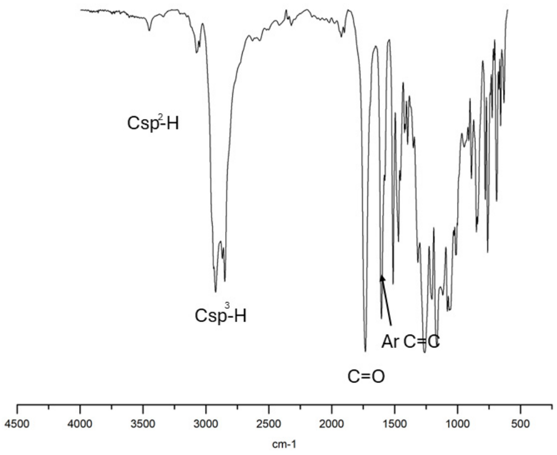

Fig. S11: *FTIR (KBr) spectrum of compound* ***TEG-Bi-11***

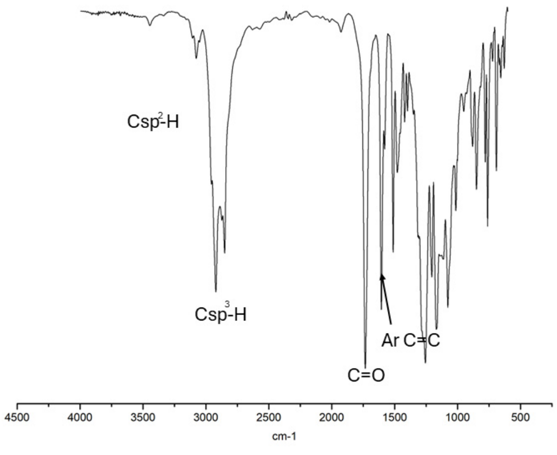

Fig. S12: *FTIR (KBr) spectrum of compound* ***11-B1-TEG***

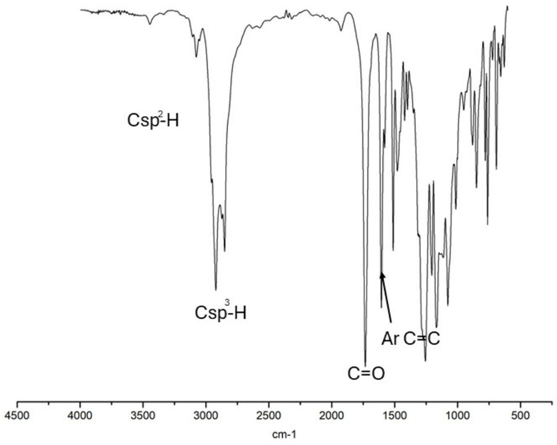

Fig. S13: *FTIR (KBr) spectrum of compound* ***TEG-B1-11***

5. HRMS Spectra

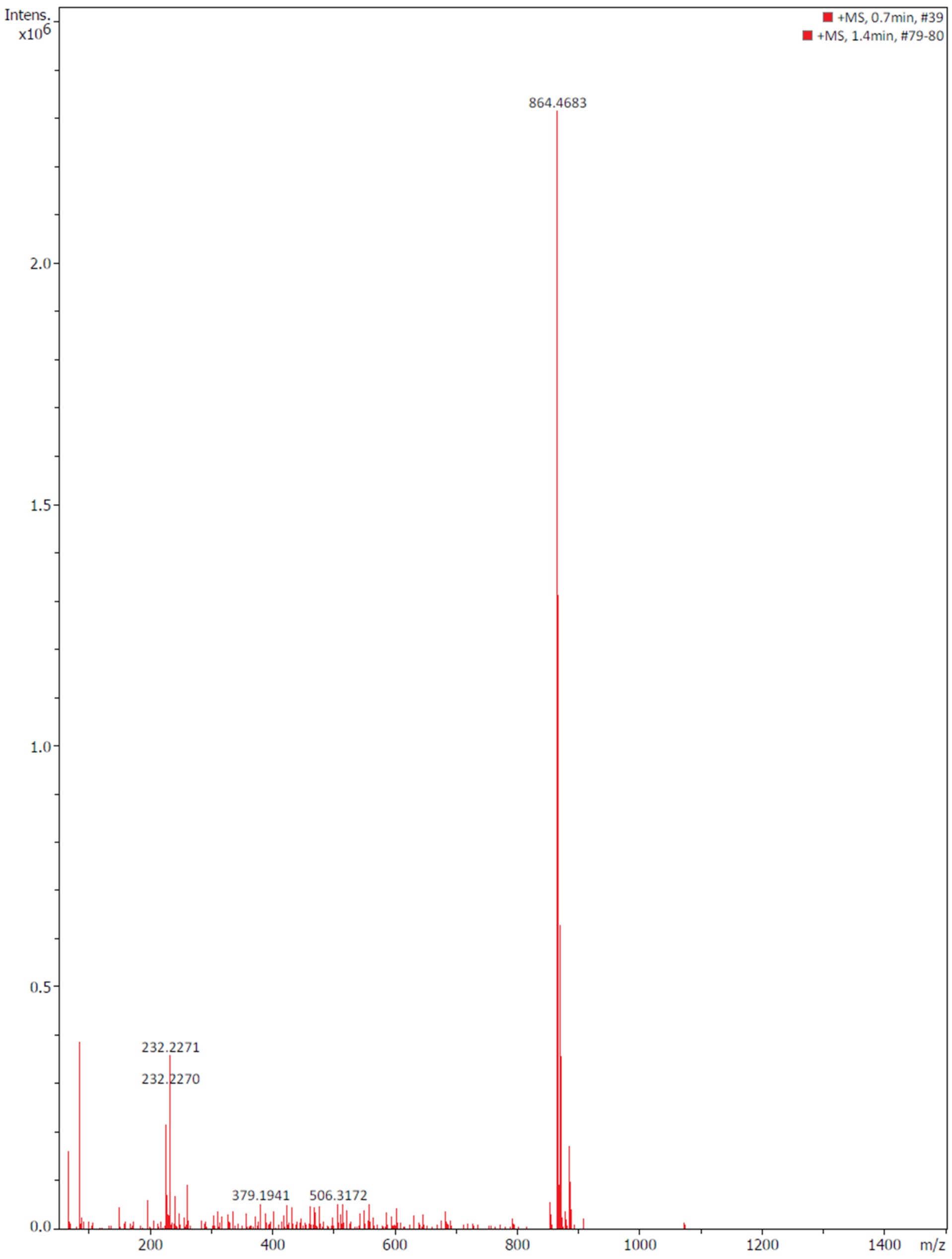

Fig. S14: *ESI+ HRMS of compound* ***11-Bi-TEG***

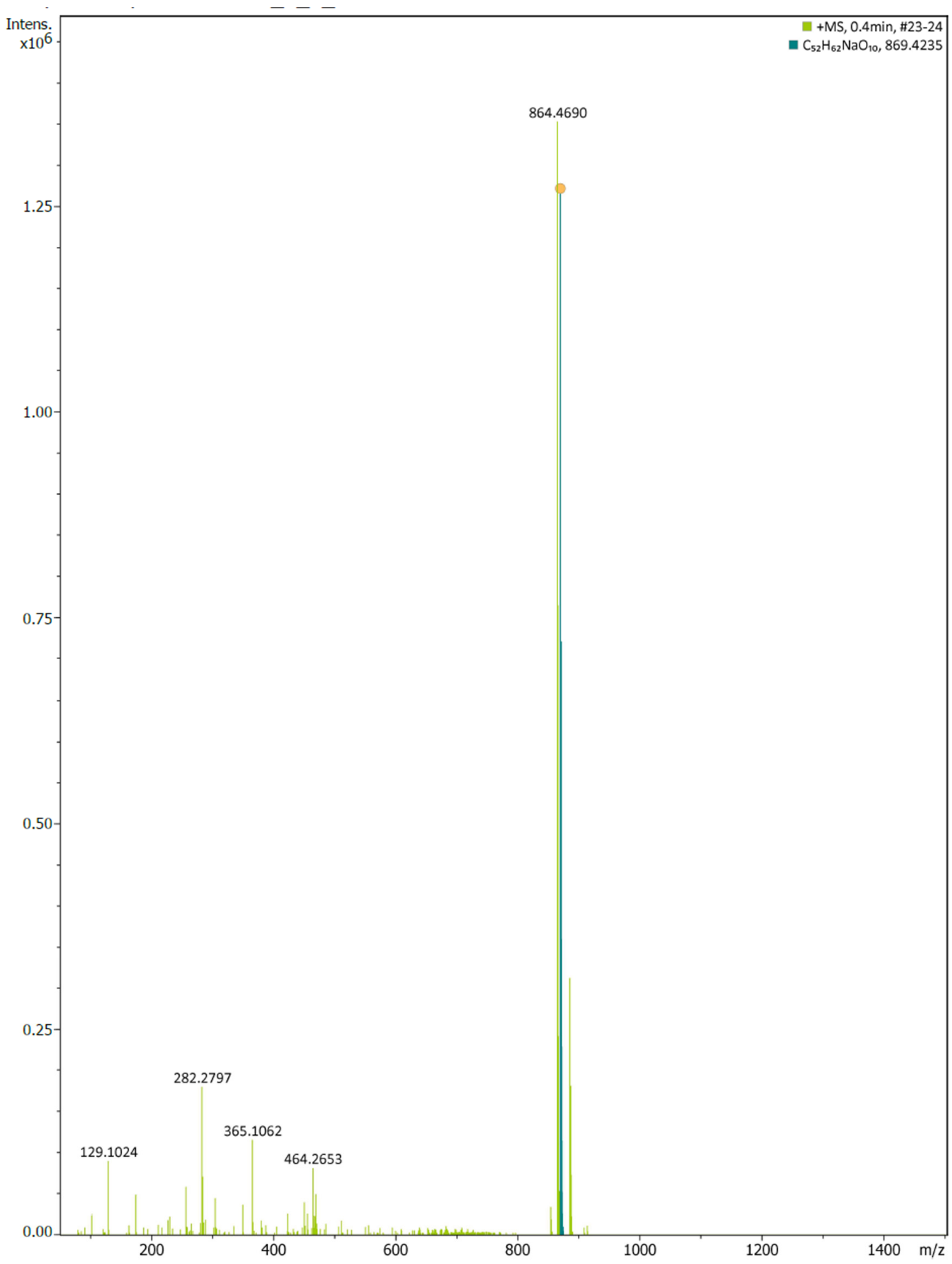

Fig. 15: *ESI+ HRMS of compound* ***TEG-Bi-11***

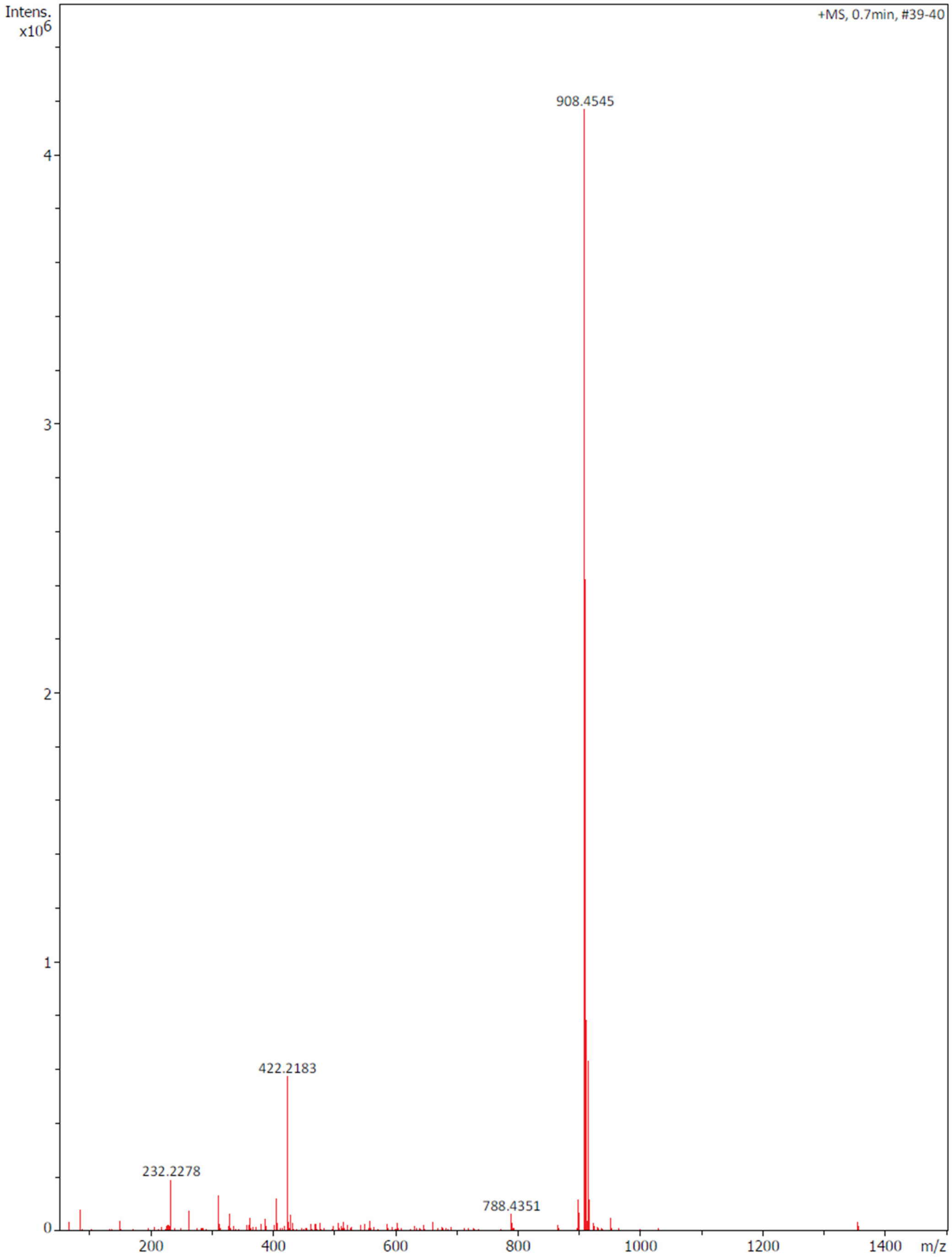

Fig. S16: *ESI+ HRMS of compound* ***11-B1-TEG***

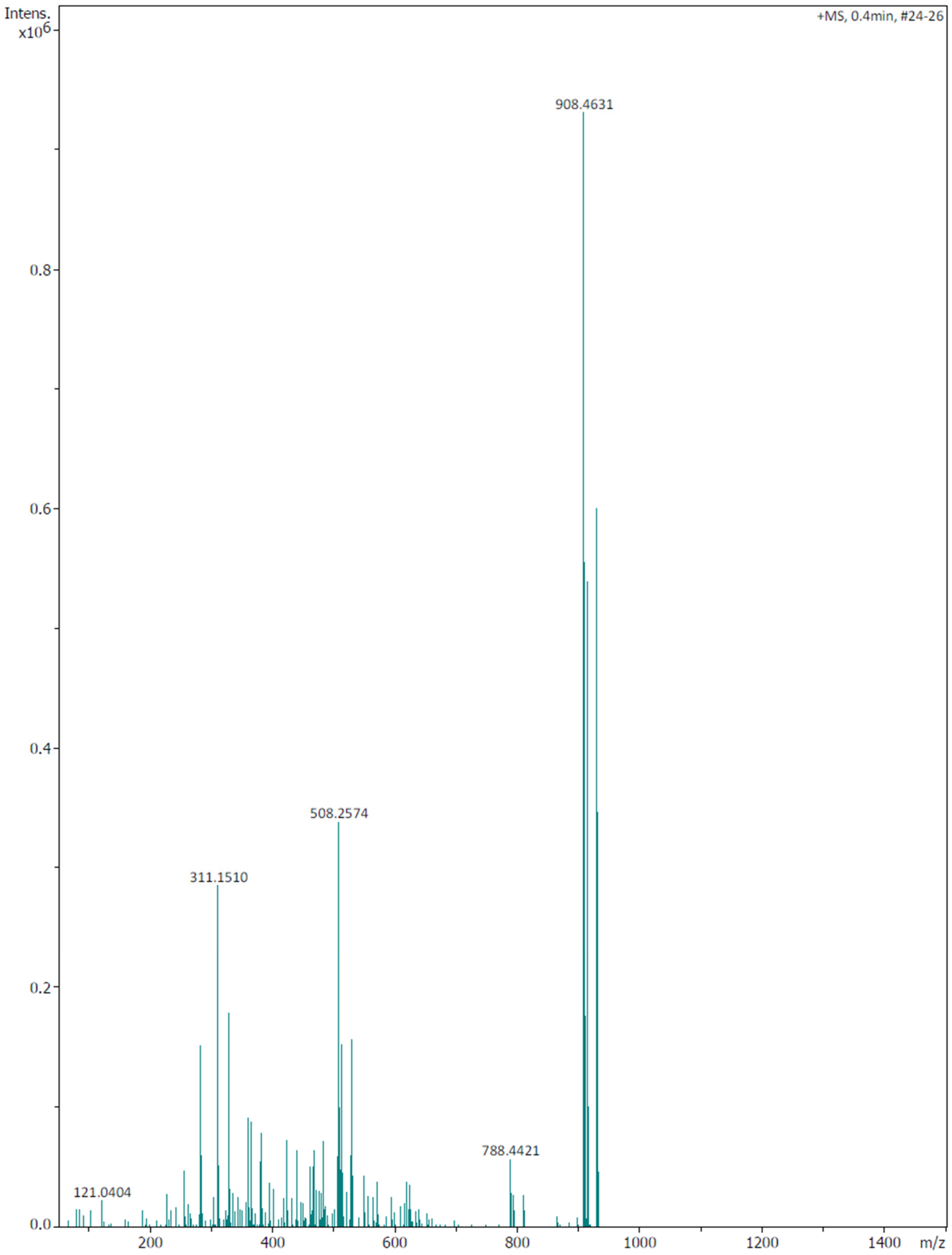

Fig. S17: *ESI+ HRMS of compound* ***TEG-B1-11***

6. Absorption Spectra

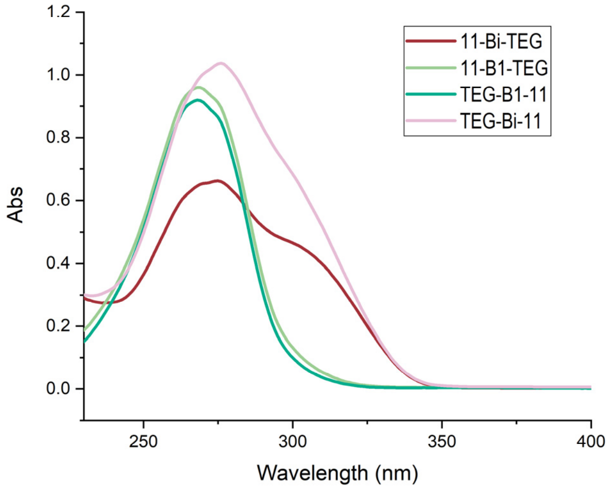

Fig. S18: *UV-Vis absorption spectra*

7. Fluorescence Spectra

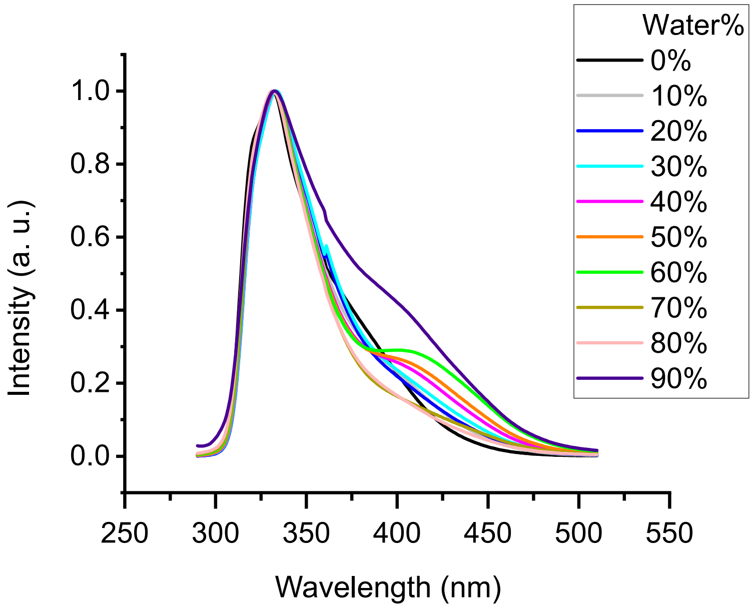

Fig. S19: *Normalized emission spectra of the compound* ***TEG-Bi-11*** *in THF with increasing additions of water, exciting at 267nm.*

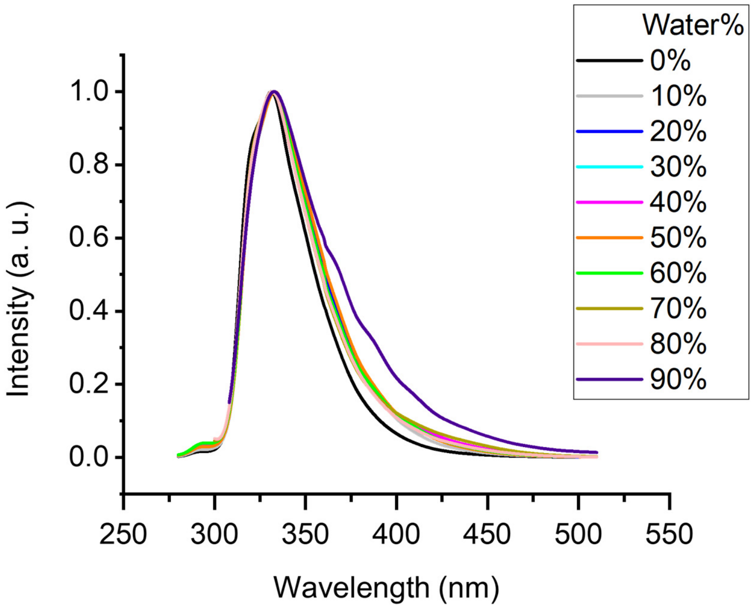

Fig. S20: *Normalized emission spectra of the compound* ***11-B1-TEG*** *in THF with increasing additions of water, exciting at 275nm.*

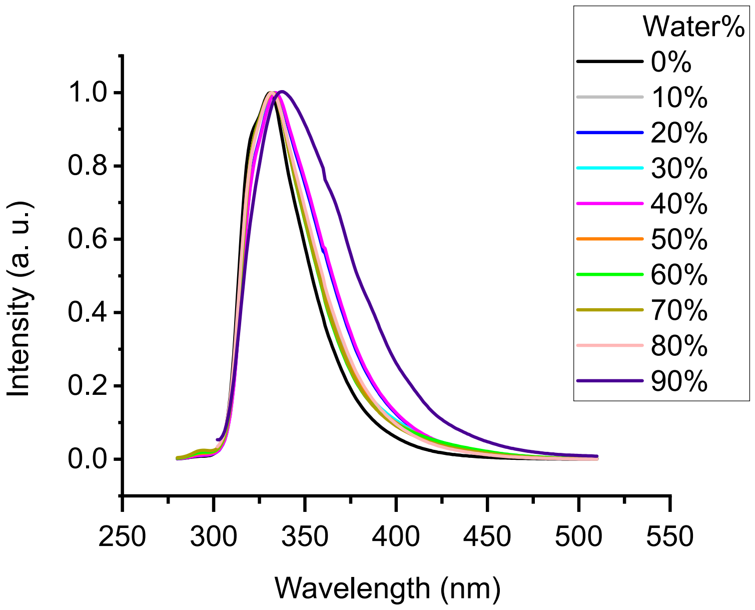

Fig. S21: *Normalized emission spectra of the compound* ***TEG-B1-11*** *in THF with increasing additions of water, exciting at 275nm.*

8.TEM Characterization

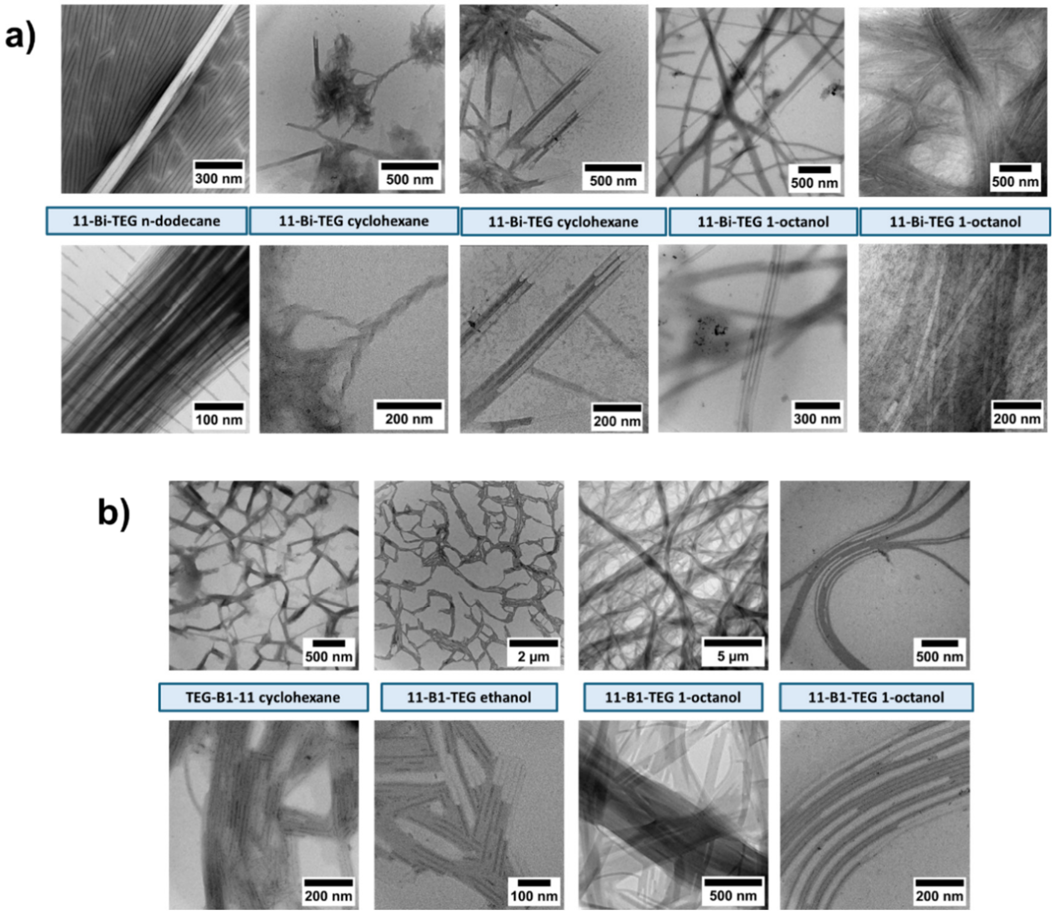

Fig. S22: *TEM pictures of fibres forming the organogels:* ***a) 11-Bi-TEG*** *and* ***b) 11-B1-TEG***

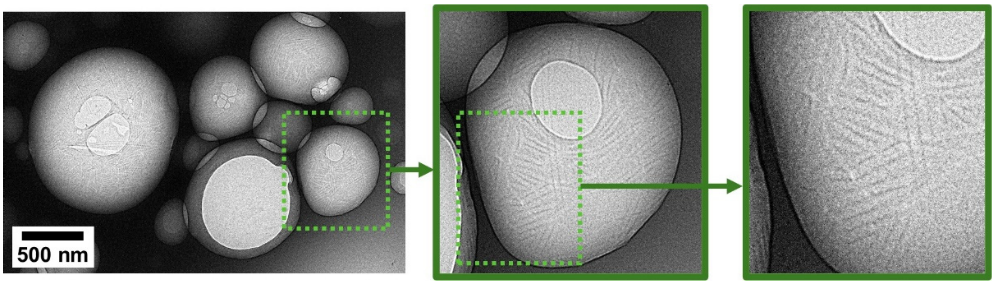

Fig. S23: *TEM images that the complex microgels preserve the dense internal fibrillar/tubular supramolecular network.*

9. XRD Pattern

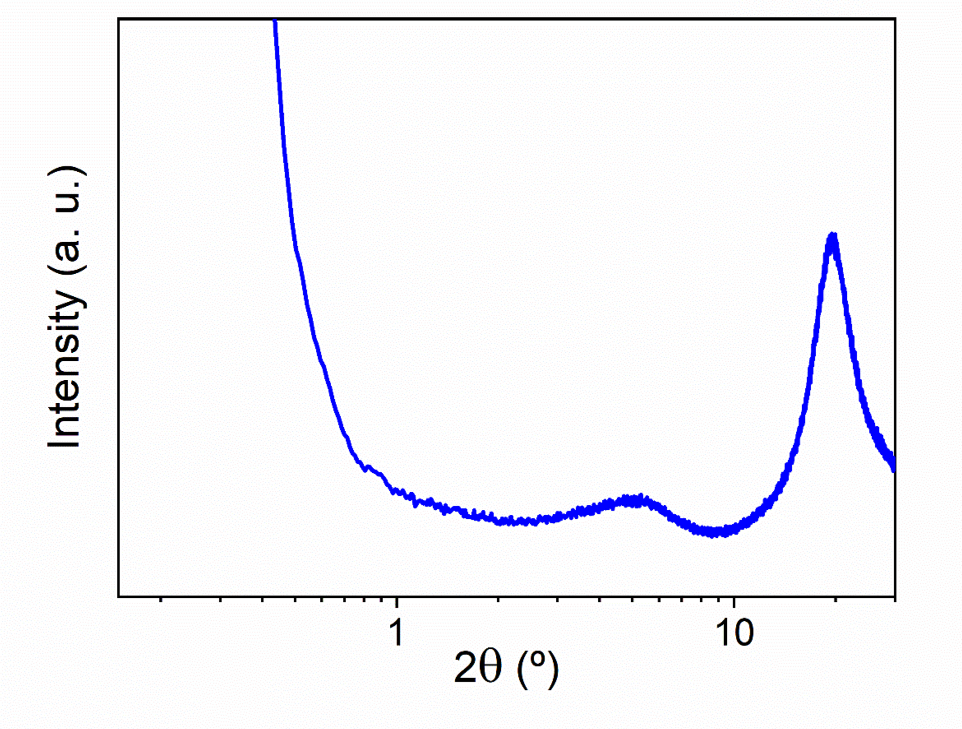

Fig. S24: *XRD Pattern of 1-octanol solvent*

10. Supplementary Figures and Tables

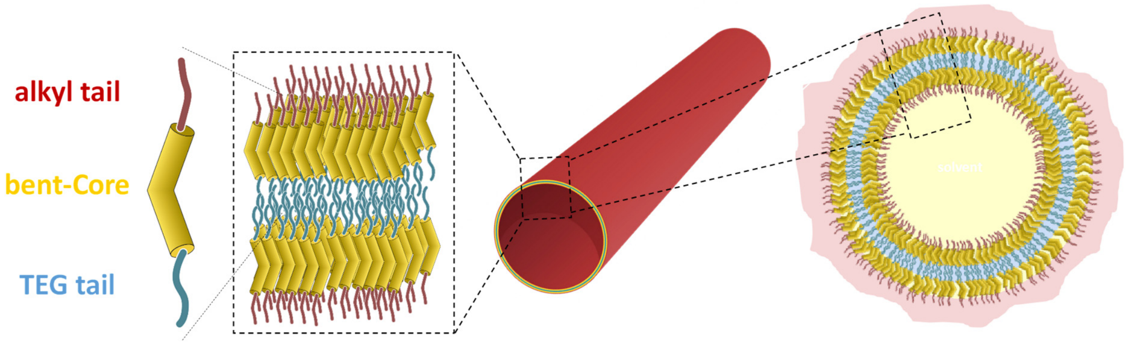

Fig. S25: *Representative schematic representation of the bilayer organization of the amphiphilic molecules proposed that constitute the network of organogels formed by the TEG-based bent-core amphiphiles studied, herein by hollow tubes (Adapted from J. Mol. Liq., 381 (2023) 121825.)*

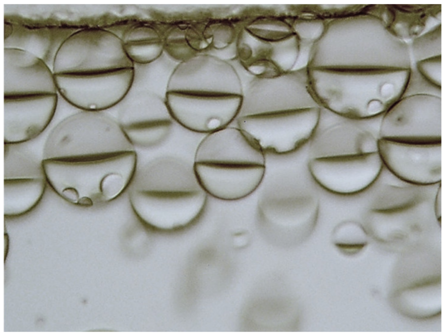

Fig. S26: *Side-view optical microscopy images of complex microgel emulsions with a Janus morphology.*

Table S1: *Morphology and dimensions of fibres forming the gels of the BC-amphiphilic compounds (w: width of fibres; L: length of fibres; Measured from TEM pictures.)*

Compound	Solvent	Morphology	Dimensions
**11-Bi-TEG**	n-dodecane	helical nanofibres	L: several µm
			w: 20-30nm
	cyclohexane	helical nanofibres	L: several µm
			w:20nm individual
		tubular nanofibres	L: less than 1 µm
			w:25-30nm
	1-octanol	tubular nanofibres	L: several µm
			w: 15-25nm
	THF/H_2O	helical nanofibres	L: several µm
			w: 25-40nm
**11-B1-TEG**	1-octanol	tubular nanofibres	L: several µm
			w: 70-150nm
	ethanol	tubular nanofibres	L: 230-330nm
			w: 10-20nm
**TEG-B1-11**	cyclohexane	tubular nanofibers	L: several µm
			w:10-20nm

11. Supplementary Videos

Video S1. Optical microscopy video recorded at 50 °C. A drop of 1 wt% Zonyl fluorosurfactant solution is introduced into an aqueous dispersion of organogel/fluorocarbon/water double emulsions stabilized with 1 wt% SDS. As Zonyl diffuses into the system and establishes a surfactant concentration gradient, the droplet morphology undergoes a dynamic transition from an organogel/fluorocarbon/water core-shell structure to a spherical Janus configuration.

Video S2. Optical microscopy video recorded at 50 °C. A drop of 1 wt% Zonyl fluorosurfactant is added to an aqueous dispersion of 1 wt% SDS-stabilized organogel/fluorocarbon/water double emulsions. Upon diffusion of Zonyl and the resulting evolution of interfacial conditions, the droplets first transform into a spherical Janus morphology and subsequently invert to form fluorocarbon/organogel/water double emulsions.

Video S3. Optical microscopy video recorded at 50 °C. A drop of 1 wt% Zonyl fluorosurfactant is added to an aqueous dispersion of Janus emulsions stabilized with a mixed surfactant system (1 wt% SDS:Zonyl, 8:2). As the local surfactant composition changes due to Zonyl diffusion, the droplets dynamically reorganize from a Janus morphology into fluorocarbon/organogel/water double emulsions.

12. References

(1) Tsai, E.; Richardson, J. M.; Korblova, E.; Nakata, M.; Chen, D.; Shen, Y.; Shao, R.; Clark, N. A.; Walba, D. M. A Modulated Helical Nanofilament Phase. *Angewandte Chemie International Edition* **2013**, *52* (20), 5254-5257. DOI: https://doi.org/10.1002/anie.201209453.

(2) Castillo-Vallés, M.; Folcia, C. L.; Ortega, J.; Etxebarria, J.; Blanca Ros, M. Self-assembly of bent-core amphiphiles joining the ethylene-oxide/lithium ion tandem. *Journal of Molecular Liquids* **2023**, *381*, 121825. DOI: https://doi.org/10.1016/j.molliq.2023.121825.

(3) Gimeno, N.; Ros, M. B.; Serrano, J. L.; de la Fuente, M. R. Hydrogen-bonded banana liquid crystals. *Angew. Chem. Int. Ed.* **2004**, *43*, 5235-5238.

(4) Nakamura, S. Liquid-crystalline compound, electrolyte containing it, and secondary battery. JP2003206291, 2003.

(5) Kovářová, A.; Světlík, S.; Kozmík, V.; Svoboda, J.; Novotná, V.; Pociecha, D.; Gorecka, E.; Podoliak, N. Unusual polymorphism in new bent-shaped liquid crystals based on biphenyl as a central molecular core. *Beilstein Journal of Organic Chemistry* **2014**, *10*, 794-807. DOI: 10.3762/bjoc.10.75.

(6) Pérez-Gregorio, V.; Cano, M.; Gascón, I.; Gimeno, N.; Ros, M. B.; Carmen López, M. Study of an ethylene oxide-terminated bent–core compound: Synthesis and Langmuir–Blodgett film structure. *Journal of Colloid and Interface Science* **2013**, *406*, 60-68. DOI: https://doi.org/10.1016/j.jcis.2013.05.074.

(7) Sletten, E. M.; Swager, T. M. Fluorofluorophores: Fluorescent Fluorous Chemical Tools Spanning the Visible Spectrum. *Journal of the American Chemical Society* **2014**, *136* (39), 13574-13577. DOI: 10.1021/ja507848f.
